# Attenuating Pregnancy Weight Gain—What Works and Why: A Systematic Review and Meta-Analysis

**DOI:** 10.3390/nu10070944

**Published:** 2018-07-22

**Authors:** Ruth Walker, Christie Bennett, Michelle Blumfield, Stella Gwini, Jianhua Ma, Fenglei Wang, Yi Wan, Helen Truby

**Affiliations:** 1 Department of Nutrition and Dietetics and Food, School of Clinical Sciences, Monash University, Clayton VIC 3168, Australia; ruth.walker@monash.edu (R.W.); christie.bennett@monash.edu (C.B.); michelle.blumfield@monash.edu (M.B.); 2 Department of Epidemiology and Preventive Medicine, School of Public Health and Preventive Medicine, Monash University, Melbourne VIC 3004, Australia; stella.gwini@monash.edu; 3 Institute of Nutrition and Food Hygiene, School of Public Health, Lanzhou University, Lanzhou 730000, China; nancymazhou@hotmail.com; 4 Department of Food Science and Nutrition, Zhejiang University, Hangzhou 310058, China; fengleiwang@g.harvard.edu (F.W.); yiwan@zju.cn (Y.W.)

**Keywords:** interventions, gestational weight gain, pregnancy, maternal, lifestyle, physical activity, diet

## Abstract

Excessive maternal gestational weight gain (GWG) contributes to generational obesity. Our aim was to explore efficacy and intervention characteristics (trimester, duration, frequency, intensity, and delivery method) of interventions to prevent excessive GWG. CINAHL, Cochrane, EMBASE, LILACS, MEDLINE, PsycINFO, and Scopus were searched up to May 2018 (no date or language restrictions). Keywords and MeSH terms for diet, GWG, intervention, lifestyle, maternal, physical activity, and pregnancy were used to locate randomized-controlled trials (RCTs). The Cochrane Collaboration tool for assessing risk of bias was applied. Eighty-nine RCTs were included. Meta-analysis (60 trials) estimated that women in diet only (WMD: −3.27; 95% CI: −4.96, −1.58, *p* < 0.01), physical activity (PA) (WMD: −1.02; 95% CI: −1.56, −0.49, *p* < 0.01), and lifestyle interventions (combining diet and PA) (WMD: −0.84; 95% CI: −1.29, −0.39, *p* < 0.01) gained significantly less weight than controls. The three eHealth interventions favored neither intervention nor control (WMD: −1.06; 95% CI: −4.13, 2.00, *p* = 0.50). Meta-regression demonstrated no optimal duration, frequency, intensity, setting, or diet type. Traditional face to face delivery of weight management interventions during pregnancy can be successful. Delivery via eHealth has potential to extend its reach to younger women but needs further evaluation of its success.

## 1. Introduction

Excessive maternal gestational weight gain (GWG) contributes to the global obesity epidemic and is a serious public health concern [1,2]. Criteria set by the United States’ Institute of Medicine (IOM) in 2009 [3], which are based on maternal pre-conception body mass index (BMI), have been adopted to define optimal GWG, with recommended GWG being lower for those of a higher BMI than for their thinner counterparts. Approximately 40–70% of women gain in excess of the IOM recommendations [2,4,5,6] with those most at-risk being already overweight or obese at conception [5]. The generational impact of obesity continues as those who experience excessive GWG are more likely to retain weight post-partum, enter subsequent pregnancies in a higher weight category [7,8], and their offspring are more likely to become overweight or obese [8,9,10,11]. Excessive GWG increases the risk of pregnancy complications such as gestational diabetes mellitus (GDM) [12] and hypertension [13] and infants are at a greater risk of being large for gestational age (LGA) (weight > 90th percentile), delivered by caesarean section [10,14,15], or experience birth trauma [10,16]. Additionally, a mother’s nutrition, lifestyle, and weight-gain during pregnancy impacts on fetal gene expression in utero and creates a ‘health blue-print’ that determines a child’s predisposition to chronic disease later in life [1,17]. 

There is no doubt that innovative and cost-effective interventions that prevent excessive GWG are required at the community level [15], particularly for women who are already overweight or obese [18,19]. Previous systematic reviews have proved effectiveness of lifestyle interventions based on altering diet and/or physical activity (PA) in clinical and community settings [19,20,21,22,23,24,25], but the heterogeneity of interventions, small sample sizes, and inconsistent results between women of different weight categories has made it impossible to define what makes an “ideal” intervention [20,26]. Interventions that are proven effective in clinical trials generally fail to be translated effectively into the real world [27]. Emerging use of innovative eHealth models that deliver information through the Internet or related technology [28] may assist in the engagement of younger women [29,30], but it is unknown whether addressing other lifestyle factors such as sleep may be helpful additive strategies. To date, there is no systematic review of all randomized-controlled trials (RCTs) aimed at preventing GWG that include all potentially modifiable lifestyle behaviors (including sleep) and medical interventions or using eHealth. Furthermore, individual components of interventions, such as duration and frequency, have not been evaluated in order to provide insight into specific characteristics that are more likely to result in success [26,31]. The aims of this systematic review and meta-analyses were to address these gaps by comparing the efficacy of all approaches that have been used to prevent excessive GWG (e.g., diet, PA, lifestyle, sleep, eHealth, and medical) and to further describe and explore characteristics that can be analyzed across intervention types such as, when they commenced (trimester), duration (weeks), frequency (occasions of contact with those delivering the intervention), intensity (hours), and delivery method (individual, group, or mixed). This knowledge would enable practitioners to have a tool box of ‘best bets’ in terms of what works that they can apply directly into practice settings.

## 2. Materials and Methods 

The review protocol was developed using the Preferred Reporting Items for Systematic Reviews (PRISMA) guidelines and registered on 29 February 2016 on the PROSPERO database for systematic reviews (PROSPERO 2016:CRD42016035907). This review reports on the primary outcomes only. The secondary outcomes are reported elsewhere [32].

### 2.1. Sources

A systematic search was conducted in April 2016 and updated in May 2018 by two independent reviewers. Electronic databases were MEDLINE, EMBASE, Cochrane Library, PsycInFO, CINAHL, Scopus, and LILACS. The search strategy (Table S1) of keywords and MeSH terms was adapted to each database. Keywords were searched as free text in title, heading, and subheading with no date or language limits. Additional publications were identified from searching reference lists of included trials and systematic reviews.

### 2.2. Study Selection

The search located RCTs of all interventions designed to prevent excessive GWG. Interventions that had GWG as a secondary outcome were also included. Comparators were standard care, an alternate intervention, or placebo. Participants were women of all ages, ethnicities, and pre-pregnancy weight status with singleton pregnancies. Studies specifically designed for women with pre-existing diabetes (type 1 or type 2) or GDM were not included due to the variation in their nutritional and medication requirements. 

Following the removal of duplicates, two reviewers independently assessed each study for eligibility based on title and abstract. Eligible studies were reviewed in full text and subjected to a second assessment for inclusion by two reviewers. Discrepancies were resolved by a third independent reviewer. If there were multiple reports of a single study, the publication that reported GWG as a primary outcome was included. 

Mean GWG (kg) with standard deviation for intervention and control groups, characteristics of participants and the interventions (when they commenced, duration, frequency, intensity, and setting) were extracted from each study. Missing information was sought from primary authors. 

### 2.3. Assessment of Risk of Bias in Included Studies

The Cochrane Collaboration tool for assessing risk of bias [33] was used to evaluate seven domains: random sequence allocation; allocation concealment; blinding of participants and personnel; blinding of outcome assessment; incomplete outcome data; selective reporting; other bias. Bias was classified as being “low risk”, “high risk‘’, or “unclear” according to predetermined criteria set by the Cochrane Collaboration [33]. Two reviewers per paper independently assessed the risk of bias with discrepancies resolved through discussion or the input of a third reviewer. All studies were included in the initial analyses; however studies identified as being “high risk” were removed from the sensitivity analyses.

### 2.4. Statistical Analyses

Studies were categorized into one of five groups: (i) diet, (ii) PA, (iii) lifestyle (a combination of PA and diet), (iv) eHealth, and (v) other (interventions specifically targeting adolescents, drug-based interventions, and interventions that could not be classified into any of the groups). Consistent with previous systematic reviews where interventions aimed at preventing excessive GWG types were classified into groups [20,22]. Despite eHealth interventions incorporating aspects of diet and PA, they were treated as a separate group because of their unique delivery characteristics and in an effort to keep heterogeneity within groups to a minimum. Meta-analyses were only possible for certain groups and were conducted for diet, PA, lifestyle, and eHealth interventions. Meta-analysis was not possible for studies classified as ‘other’ because of the vast differences between their designs. Studies were excluded from the meta-analyses for no true control [34,35,36,37,38,39,40,41,42,43,44] or insufficient data [45,46,47,48,49,50,51,52]. Due to different definitions and measurements for GWG, GWG was classified into sub groups (i) >24 weeks, (ii) 12–24 weeks, and (iii) <12 weeks.

High degrees of heterogeneity were expected among studies so the DerSimonian and Laird [53] approach for random-effects models was used to compute weighted mean differences (WMD) (the pooled average difference based on the size/weight of each trial) and 95% confidence intervals (CI). Heterogeneity was evaluated using the *I*^2^ statistic. Funnel plots with Egger’s test were used as a visual aid to detect publication bias [33,53,54]. Sensitivity analyses were conducted to investigate the influence of a single study on the overall analyses. 

Meta-regressions used six study characteristics as covariates to explain the heterogeneity between studies and explore whether study characteristics had a relationship with the effect sizes [55]. Study characteristics were based on a previous review [56] and included (i) the trimester in which the intervention commenced, (ii) duration (weeks—if a range the longest duration was used), (iii) frequency (one to three, four to eight, and >eight occasions of contact), (iv) intensity (<four, four to eight, and >eight hours of contact over the intervention), (v) delivery method (individual, group, and combination), (iv) diet (healthy eating advice, low-glycemic index (GI), calorie restricted, and pro-biotic).

In order to further compare the characteristics of interventions, studies were classified as being either effective or ineffective. This assessment was made by a relatively crude assessment of GWG results. Studies were classified as being effective if the intervention group had significantly less overall or weekly GWG than the control (*p* < 0.05). While this is not an ideal measure, this method has been used in previous systematic reviews [56,57,58]. The Chi-square test for independence was used to compare the characteristics of effective and ineffective studies with a statistically significant difference defined as *p* < 0.05. All meta-analyses and meta-regression were carried out using Stata 12 statistical software [59].

## 3. Results

**Figure 1:**
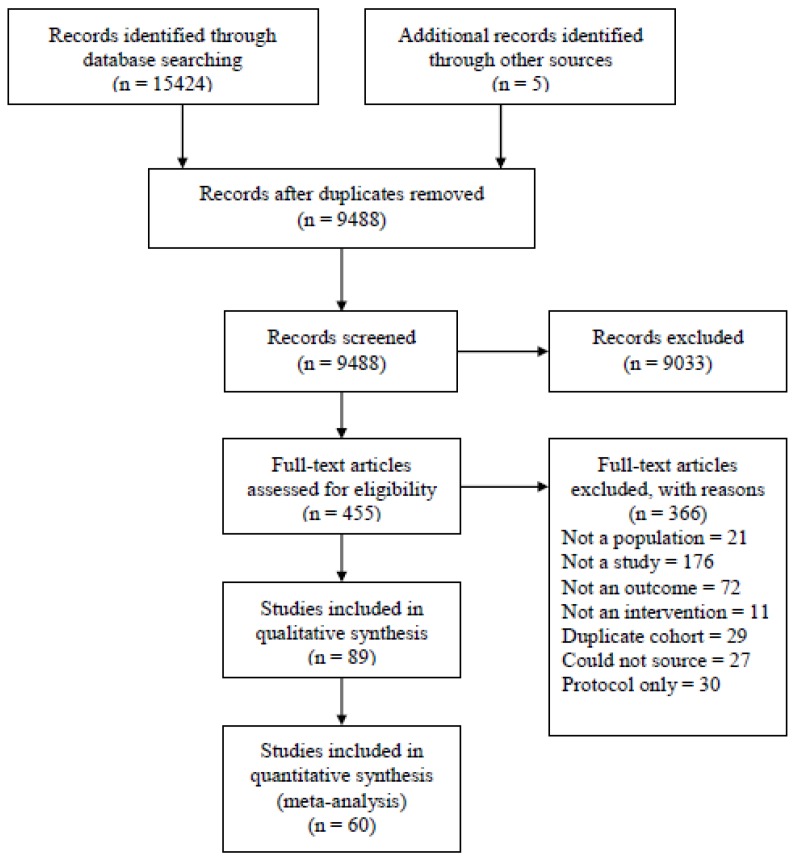
PRISMA flowchart of study selection.

Data were extracted from 89 published RCTs that included 25,345 women (Figure 1). Studies reported on singleton pregnancies in women of all BMIs (*n* = 51), healthy weight only (*n* = 3), healthy weight and overweight (*n* = 1), overweight and obese only (*n* = 21), or obese only (*n* = 13). Studies were carried out in samples of women from 24 countries the United States (*n* = 22), Australia (*n* = 11), Spain (*n* = 10), and Italy (*n* = 5) being most highly represented. Studies did not consistently report exclusion criteria for age, but those that did tended to exclude women >45 years, nor did they consistently report on baseline characteristics for SES or ethnicity. No study focused on optimizing sleep quality or duration in order to prevent excessive GWG. 

### 3.1. Diet Interventions

Sixteen diet only studies reported a total of 3681 participants (Table S2). Four interventions delivered basic healthy eating advice [46,47,60,61], four were based on a low-GI diet [36,38,44,62], and six involved calorie-restriction [37,63,64,65,66,67]. Ilmonen et al. [68] delivered dietary advice with a probiotic and Bosaues et al. [51] advised women to increase their fish intake in addition to general dietary advice. All studies commenced in the first or second trimester, had varying degrees of intensity and frequency and were delivered to women individually except for Walshe et al. [62] who delivered their intervention to both individuals and groups (Table S3). Nine diet interventions [60,61,62,63,64,65,66,67,68] were included in the meta-analyses (Table 1) (Figure S1). McCarthy et al. [61] did not report overall GWG for the entire intervention and control groups, therefore the GWG for sub-groups and their controls were used instead. The meta-analysis showed that dietary interventions were more effective than the control groups (WMD: −3.27; 95% CI: −4.96, −1.58, *p* < 0.01; *I*^2^ = 92.8%). Meta-regression revealed none of the study characteristics significantly influenced the pooled effect size (Table 2).

### 3.2. Physical Activity

Twenty-seven PA only studies reported on 5725 participants (Table S4). The PA prescriptions included light to moderate aerobic, muscle strength, toning, and flexibility exercises, or dancing, walking, or aquatics. Nine studies had relatively long durations (≥30 weeks), 24 had high levels of frequency and intensity in comparison with diet and lifestyle interventions, and most described interventions with an opportunity for women to have 80–85 occasions of contact (Table S3). Twenty-four PA interventions [69,70,71,72,73,74,75,76,77,78,79,80,81,82,83,84,85,86,87,88,89,90,91,92] were included in the meta-analyses (Table 1) (Figure S2). Meta-analysis showed that women in PA interventions gained significantly less weight than those in the control groups (WMD: −1.02; 95% CI: −1.56, −0.49, *p* < 0.01; *I*^2^ = 81.9%). Intervention effectiveness was significantly higher in studies that combined individual and group-delivery of interventions, compared with studies that were delivered to individuals only or groups only (Table 2).

### 3.3. Lifestyle Interventions (Combining Both Diet and PA), Delivered Face-to-Face

Thirty-three lifestyle studies reported on 9201 participants (Table S5). Key aspects of interventions were general healthy eating advice, 30–60 min of PA on most days, and self-monitoring. Most lifestyle interventions had a duration of 13–29 weeks with varying frequencies and intensities (Table S3). Twenty-four lifestyle interventions [93,94,95,96,97,98,99,100,101,102,103,104,105,106,107,108,109,110,111,112,113,114,115,116] were included in the meta-analyses (Table 1) (Figure S3). Four studies [94,95,102] did not report overall GWG for the entire intervention and control groups, therefore the GWG for weight sub-categories based on BMI were used. Bogaerts et al. [104] and Guelinckx et al. [99] included two intervention groups with one control group. For these studies, the more intensive intervention (lifestyle advice and a brochure) was chosen for meta-analysis. Results from the meta-analysis favored LS interventions over comparators (WMD: −0.84; 95% CI: −1.29, −0.39, *p* < 0.01; *I*^2^ = 71.0%). Meta-regression revealed no significant differences across studies observed by any study characteristic (Table 2). 

### 3.4. eHealth Interventions

Five studies reporting on 2168 women incorporated aspects of information technology, eHealth or mHealth (use of mobile device) (Table S6). Herring et al. [117] and Willcox et al. [118] provided regular health coaching regarding lifestyle, physical activity, and GWG to women via Facebook, text-messaging and/or a website, booklets, and phone-calls. Olsen et al. [119] and Smith et al. [120] based their interventions on websites and Jackson et al. [121] described an intervention that added ‘Video Doctor’ counseling in addition to their routine antenatal care (Table S3). Three eHealth interventions [117,118,120] were included in a meta-analysis which favored neither intervention nor control (WMD: −1.06; 95% CI: −4.13, 2.00, *p* = 0.50; *I*^2^ = 73.6%) (Table 1) (Figure S4). 

### 3.5. ‘Other’ Interventions

Eight studies reporting on 1906 women were classified as “other” (Table S7). Four interventions were based on regular weighing [30,122,123,124] however, only Quinlivan et al. [124], incorporating continuity of care and dietary counseling reported a significant difference between intervention and control. Syngelaki et al. [29] compared metformin with placebo in pregnant women without GDM, Bechtel-Blackwell et al. [30] described an intervention designed for 46 African-American pregnant adolescents and Santamaria et al. [125] explored whether myo-inositol supplementation could decrease the incidence of GDM in women who were overweight. Herrera-Perdigon et al. [126] conducted a secondary analysis of an RCT in high-risk pregnancies where every second visit in usual physician-led care was substituted with home-based visits from advanced practice nurses. The characteristics of ‘other’ studies varied with only four [29,117,118,124] reporting significantly less GWG in the intervention groups (Table S3). 

### 3.6. Effectiveness of Interventions According to Their Characteristics

Of the 89 studies included, 73 could be classified as being either effective (32 studies) or ineffective (41 studies) (Table 3). Studies could not be classified because of no overall *p* value to classify them as effective or ineffective [47,94,95,102,127] or not having a true control [34,35,36,37,38,39,40,41,42,43,44]. Interventions delivered to women individually were significantly more likely to be ineffective (ineffective 66.7%, effective 33.3%; *χ*^2^ 4.43 *p* = 0.04). In contrast, when combining interventions delivered to groups and those delivered to individuals with a group component involved, a significantly larger proportion were effective (effective 62.5%, ineffective 37.35%; *χ*^2^ 5.06 *p* = 0.02).

### 3.7. Risk of Bias 

**Figure 2:**
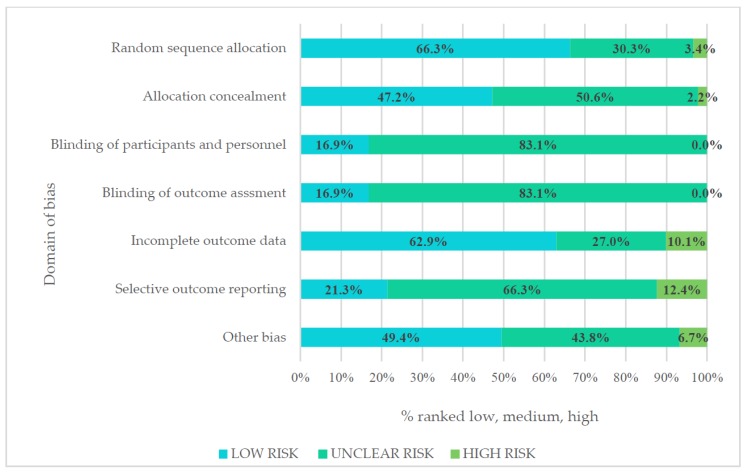
Risk of bias assessed using the Cochrane collaboration tool for assessing risk of bias.

A high risk of bias was allocated across domains and overall, 24% (*n* = 21) of interventions had a high risk of bias (Figure 2). Most studies had an unclear risk of bias for blinding participants, personnel, and outcomes as it was not possible to definitively assess whether minimal blinding inherent in dietary and lifestyle interventions impacted on outcomes. Studies were allocated either unclear or high for selective reporting as they did not have a published protocol, or they did not report on all outcomes. This was particularly true for older publications.

To explore whether the high level of asymmetry displayed in the funnel plots (Figures S5–S8) was due to publication bias, selective outcome reporting, and/or inadequate analysis in individual studies [128], studies with a high risk of bias were removed. There was slightly less asymmetry in the GDM and eHealth funnel plots only, a potential explanation being the considerable differences in characteristics (i.e., intensity) of studies included in each meta-analysis [128]. There were too few studies in the GDM and eHealth meta-analyses to differentiate between chance and true asymmetry [128]. 

### 3.8. Sensitivity Analyses

The sensitivity analyses revealed that no single study introduced a large degree of bias to any of the meta-analyses. To explore whether the quality of studies, as rated by risk of bias, impacted on the meta-analyses, all studies with a high risk of bias were removed. This resulted in slight reductions in the heterogeneity of all meta-analyses except for diet and PA (Table S8). Results for LS interventions (WMD: −0.73; 95% CI: −1.17, −0.29, *p* < 0.01; *I*^2^ = 65.7%) were tempered. In contrast, the WMD of eHealth interventions shifted to favor intervention (WMD: −2.26; 95% CI: −3.84, −0.69, *p* < 0.01; *I*^2^ = 0.00%).

## 4. Discussion

This systematic review demonstrated that women exposed to interventions with a primary or secondary outcome of GWG gained less weight than control groups. This reaffirms that interventions aimed at preventing excessive GWG can be effective across a range of settings and population groups [19,20,22,24]. The evidence base regarding interventions that incorporate eHealth and information technology is small, however our findings highlight their potential to support women throughout pregnancy. Unique to this review is meta-regression that found there is no optimal duration, frequency, intensity, delivery method, or diet for interventions aiming to prevent excessive GWG, making it impossible to definitively describe a tool box of ‘best bets’ that can be applied directly into practice settings. Despite this, our synthesis of international literature provides valuable insights to guide clinical practice and the planning and implementation of future interventions that are tailored to meet the needs and expectations of women from a diverse range of cultures and social contexts. 

Rogozinska et al. [31] used individual patient level data from 33 trials to report that women who undertook any type of intervention involving diet and/or PA during pregnancy could gain 0.7 kg less than those not exposed to an intervention. Their analysis consisted of mainly white (80%), high income participants of whom 40% were obese at the time of conception and/or reported no exercise at baseline. Shifting energy balance to favor weight-loss or weight maintenance by dietary restriction is far simpler than it is by expending energy via activity [129], especially in those who are not used to undertaking planned exercise, which requires substantial shifts in behavior. Knight and Foster [130], commenting on Rogozinska’s review, suggest that it supports the safety of current diet and PA recommendations but state that it provides little evidence of the benefit of ‘lifestyle’ interventions per se. This unidirectional outlook misses the long term impact that small shifts in dietary patterns towards those with greater nutrient density can have on future generations [17], especially in those who are already obese and have very sedentary lifestyles.

### 4.1. Dietary Interventions

The meta-analyses revealed the largest WMD in diet interventions, upholding that a diet-based approach is more effective than PA or lifestyle interventions [22]. Meta-regression suggested that the type of diet, whether it be low-GI, calorie restriction, or simple healthy eating advice was not a factor that influences the outcome, however, the small data-set included in this component of the analyses should be taken in to consideration. The hypothesis that any type of healthy diet can be effective in preventing excessive GWG is supported by individual studies included in this review. Moses et al. [44] and Rhodes et al. [38] found no difference in GWG when comparing to different diet-types. It seems that any dietary improvement is better than doing nothing for the prevention of excessive GWG and simple dietary advice may be just as effective as time-consuming and labor-intensive personalized dietary regimens. Healthy eating should not be overlooked as a key pillar of antenatal care for all women, particularly when for the first time in history there are more people in the world who are overweight than underweight [131], and more than half of women enter pregnancy already overweight or obese [31,132]. 

### 4.2. Individual, Group or Mixed Delivery? 

Meta-regression did not consistently distinguish between individual and group delivery of interventions with a combined approach appearing to be more effective in PA interventions only. Intervention groups in the two eHealth studies that used Facebook as part of a multifaceted approach [117,118] had significantly less GWG than controls. In contrast, intervention groups in eHealth interventions using websites or information technology with no opportunity for women to interact with each other did not gain significantly less weight than control groups. Comparison of intervention effectiveness according to characteristics (Table 3) found a significantly larger proportion of interventions that had a group element were effective than ineffective. This has important implications for future interventions and their translation in real-world settings. Interventions delivered to groups of women have the potential to reach a wider audience in communities at a lower cost [133,134]. There are also aspects of group-based interventions upon which value cannot be placed or measured. These include emotional and social support, peer-contribution to learning, affirmation, and a sense of ‘togetherness’ that increases womens’ motivation to achieve their health goals [134]. It should be emphasized that interventions are likely to have a greater impact on population incidence of overweight and obesity if sub-groups most at-risk for excessive GWG, such as women who are already overweight and obese, low socioeconomic status (SES), and certain ethnic minorities are targeted [133].

### 4.3. Intensity and Frequency of Interventions

Neither the frequency nor intensity of interventions aimed at preventing excessive GWG had a consistent influence on the overall effect size of interventions. There is accumulating evidence from individual trials suggesting that interventions with low levels of intensity and frequency can be successful. The HeLP-her cluster RCT [135] found that weight gain could be prevented in women living in metropolitan Australia, with a low intensity and frequency intervention. These findings were replicated when carried out in rural settings in Australia [136]. When the HeLP-her methodology was applied in a pregnant population at risk of GDM, it resulted in significantly less weight gain in the intervention group compared with the control [105]. This synthesis is important as it suggests that intensive and costly interventions are not essential in order to prevent excessive GWG. In the future, well-designed interventions aimed at preventing excessive GWG should include a standardized cost-effectiveness analysis so that this theory can be investigated further. 

### 4.4. Trimester and Duration of Interventions

Excessive GWG in the first trimester (in excess of 0.5–2 kg) [3] is predictive of excessive GWG throughout pregnancy [137], maternal hyperglycemia [138], and the development of GDM [18]. Therefore, preventing excessive GWG in early pregnancy is important for preventing pregnancy complications and excessive GWG overall [18,137]. This is problematic, considering that a woman’s first antenatal check-up may not occur until well into the first trimester and half of pregnancies are unplanned [139]. Therefore, public health campaigns regarding optimal nutrition and lifestyle targeted to all women of childbearing age plans are required [140]. When considering that women tend to gain approximately 500 g–1 kg per year from their early 20 s until middle-age [141], such campaigns would be of benefit to all women and not just those planning pregnancy.

### 4.5. Future Directions

Increased prevalence of being overweight or obese at conception and excessive GWG have ignited the debate regarding routine weighing by health professionals [142]. One side of the debate argues that there is ‘no point assessing weight gain if there is no way of influencing it’, [142], and that routine weighing may be a cause of unnecessary stress for women [143]. The other side argues that rates of excessive GWG are increasing and routine weighing by a health professional is a low-cost intervention that can be applied in established antenatal care settings [142,144]. Routine weighing by a health professional was a key component of four studies in this systematic review [30,122,123,124]. Interestingly, only one of these studies that also involved regular dietary counseling and continuity of care resulted in a significant difference in GWG between intervention and control [124]. An important aspect of antenatal care in relation to this issue is that pregnant women require appropriate information and advice and continuity of care in order to fully support weight management [124]. This is supported by recent qualitative research that has found that women want clear, consistent advice regarding weight management during pregnancy [145]. Barriers that hinder health professionals from providing this advice could be addressed, at least in part, by providing them with further training in how to initiate a conversation regarding GWG and then equipping them to provide consistent and patient-centered advice [139,146,147]. 

Potential assets for the prevention of excessive GWG that have been overlooked in many RCTs are established antenatal care models within the local context of intervention settings. Even simple conversations regarding weight management initiated by a health professional and integrated into routine antenatal care may be highly effective in preventing excessive GWG [26,148], not to mention adverse pregnancy outcomes associated with excessive GWG that were outside the scope of this study such as GDM [32], large for gestational age, macrosomia, and cesarean delivery [15]. Rather than developing expensive and intensive interventions as an adjunct to antenatal are, perhaps the focus should be supporting and refining pre-existing antenatal services that already receive government funding [26]. Incorporating aspects of eHealth (including mHealth) may facilitate health messages with a broader reach [149]. What makes interventions successful (or not) is rarely elucidated in RCTs, suggesting that alternate intervention designs that incorporate qualitative evaluation may be of value. 

In terms of implications for practice, the earlier dietary or lifestyle changes are adopted for pregnancy, the better. We have confirmed that dietary improvement can be effective in attenuating GWG, however, we cannot describe the optimal ‘pregnancy diet’ based on our results. This is an important reminder that dietary advice should consider an individual’s preferences, budget, lifestyle, and food preparation skills [150,151]. The value of small “nudges” towards achievable dietary and lifestyle changes from trusted health professionals [139], and the potential benefits of offering advice in a group context where women may receive encouragement and support from peers [134] should not be underestimated. Put simply, doing something is better than doing nothing.

### 4.6. Strengths and Limitations

A strength of this review is its broad general applicability across populations and population sub-groups. This is because of a robust study design that included RCTs only, with no language or date limits, from seven international databases. It is also the first review of interventions aimed at preventing excessive GWG to analyze and report on which characteristics of interventions may be more likely to influence their success.

The high levels of heterogeneity (*I*^2^ > 75%) [152] in three of the five meta-analyses is a limiting factor, an issue associated with earlier reviews on the same topic [19,22,23]. Proactive steps were taken to address this in the design of this research, such as dividing interventions into five different meta-analyses and not including ‘other’ studies. A random-effects model for meta-analysis was deemed appropriate from the outset [53] and meta-regression was embedded into the design to determine the degree to which statistical heterogeneity can be attributed to covariates (study characteristics) [53]. There were very few studies within sub-groups for each meta-regression and therefore, caution should be taken when interpreting these data. Despite high levels of heterogeneity, our conclusion that interventions designed to prevent excessive GWG can be effective, and diet interventions are most effective, is consistent with previous research [22]. 

The analysis and interpretation of these data is further limited by the inconsistent outcome measures used for GWG. Using broad categories that cluster different measurements of GWG is not ideal, however, recent research has found using an early pregnancy weight (4–10 weeks gestation) is a reasonably accurate surrogate measure for pre-pregnancy BMI [153]. While this justifies the decision to cluster pre-pregnancy and early pregnancy measurements of GWG together, it does not justify clustering together interventions of a similar duration that began at different points of pregnancy where IOM recommendations for weekly GWG are different. Some studies did not specify how they calculated GWG, requiring reviewers to make assumptions based on baseline characteristics and overall GWG. In the future, these limitations could be mitigated if interventions clearly report a standard measure for GWG and other maternal and fetal outcomes [31]. The quality of studies must also be considered in the interpretation of results. Future studies should report measures taken to ensure blinding and all outcomes outlined in registered protocols which would allow greater detail to be provided of the actual content of the intervention.

This review highlights several key areas where future research is required regarding weight management in pregnancy. Interventions should adopt universal definitions and measurements for key outcomes such as GWG and GDM, as well as standardized cost-effectiveness analyses. In order to improve the quality of studies, measures should be taken to ensure blinding and that all outcomes are outlined in registered protocols.

Other methods of assessing intervention success may need to be explored, given the high levels of heterogeneity found within ‘like’ studies that have been frequently clustered together in meta-analyses [19,22]. There may be benefit in focusing on population sub-groups most at risk of excessive GWG, and apply findings by making specific recommendations for sub-groups rather than a ‘one size fits all’ approach. Attention should also be given to reporting change in diet quality to provide more insight into micronutrient status and also metabolic outcomes pertinent to PA interventions such as strength or aerobic fitness. Qualitative methods may be required to truly understand the context, barriers, and enablers of interventions. Despite the lack of a universally accepted approach to weight management in pregnancy, it is clear that doing something is better than doing nothing. The timely translation of research into practice is crucial when considering that excessive GWG has emerged as a significant health issue for all women in low, middle, and high income countries [1,10,154].

## 5. Conclusions

This review found that interventions aimed at preventing excessive GWG can be successful, however, characteristics of interventions such as when they commence, their intensity, frequency, duration, or delivery methods are not predictors of success. This has important implications for designing future interventions that may reach more women with less expense. Refining pre-existing antenatal care models and/or incorporating some dietary aspects of all interventions could be cost-effective approaches. Some women may experience only a slight reduction in GWG as a result of the advice they receive during pregnancy. Despite this, healthy lifestyle behaviors, adopted at a time when women are highly motivated to make lifestyle changes and with the ongoing support of a health professional, have the potential to positively impact on families and households even after a child is born. 

## Figures and Tables

**Table 1:** Random-effects meta-analyses for diet, physical activity, lifestyle, and eHealth interventions (WMD and 95% CI). (Figures of meta-analyses are Supplementary Figures S1–S4).

	Sub-Group and Overall	Studies ^a^	Participants	Overall Effect Size WMD (95% CI)	*p* Value	*I* ^2^
Diet	Overall: Measurement GWG > 24 weeks	9	2049	−3.27 (−4.96, −1.58)	<0.00	92.8%
Physical activity	Measurement GWG 12–24 weeks	3	250	−0.83 (−3.55, 1.90)	0.55	83.3%
	Measurement GWG > 24 weeks	21	4651	−1.02 (−1.56, −0.47)	<0.00	81.8%
	Overall	24	4901	−1.02 (−1.56, −0.49)	<0.00	81.9%
Lifestyle	Measurement GWG 12–24 weeks	5	2908	−0.67 (−1.58, 0.23)	0.14	80.1%
	Measurement GWG > 24 weeks	19	4471	−0.92 (−1.48, −0.36)	<0.00	69.8%
	Overall	24	7379	−0.84 (−1.29, −0.39)	<0.00	71.0%
eHealth	Overall: Measurement GWG > 24 weeks	3	192	−1.06 (−4.13, 2.00)	0.50	73.6%

^a^ Some studies included sub-groups as no overall GWG was reported.

**Table 2:** Meta-regression of characteristics of diet and lifestyle interventions designed to prevent excessive GWG.

	Diet (10 Observations)				PA (25 Observations) *I*^2^ = 82.0%				Lifestyle (27 Observations) *I*^2^ = 71.0%			
Characteristic	*n*=	ß	95% CI	*p*	*n*=	ß	95% CI	*p*	*n*=	ß	95% CI	*p*
**Trimester**	**9**				**25**				**27**			
1	3		Ref		12		Ref		6		Ref	
2	6	0.35	−4.61, 5.32	0.87	13	0.81	−0.13, 1.75	0.09	21	−0.16	−1.50, 1.17	0.81
3	0	NA	NA	NA	0	NA	NA	NA	0	NA	NA	NA
**Intensity**	**10**				**25**				**23**			
1–4 h	5		Ref		5		Ref		8		Ref	
>4–8 h	2	−1.08	−7.43, 5.28	0.70	1	1.50	−2.77, 5.77	0.47	7	0.00	−1.46, 1.46	1.00
≥8 times	3	0.71	−5.04, 6.46	0.78	19	0.05	−1.37, 1.48	0.94	8	−1.32	−2.71, 0.08	0.06
**Frequency**	**10**				**25**				**27**			
1–3 times	5		Ref		3		Ref		3		Ref	
4–7 times	1	−1.92	−10.18, 6.33	0.60	2	0.55	−2.71, 3.81	0.73	10	0.18	−1.79, 2.14	0.86
≥8 times	4	0.46	−4.73, 5.65	0.84	20	0.02	−1.94, 1.98	0.99	17	−0.04	−1.95, 1.88	0.97
**Setting**	**10**				**20**				**27**			
Individual	9		Ref		7		Ref		19		Ref	
Group	0	NA	NA	NA	11	−0.62	−1.56, 0.31	0.18	8	−1.09	−2.28, 0.09	0.07
Mixed	1	2.01	−5.17, 9.20	0.54	2	−1.99	−3.45, −0.52	0.01	0	NA	NA	NA
**Duration**	**9**				**25**				**27**			
Reference			Ref				Ref				Ref	
		−0.65	−1.81, 0.52	0.23		0.01	−0.09, 0.10	0.90		−0.04	−0.12, 0.04	0.34
**Type**	**10**											
General	3		Ref									
Low-GI	1	−0.06	−8.59, 8.48	0.99								
kJ restriction	5	−3.20	−8.81, 2.42	0.21								
Probiotic	1	−2.56	−11.32, 6.21	0.50								

Note: Observations, not studies (some studies were analyzed as sub-groups). Characteristics unknown for some studies. Abbreviations: NA = none of the studies in that intervention type displayed this characteristic. Ref: Reference group.

**Table 3:** Summary of the effectiveness of interventions according to characteristics.

		Effective, *p* < 0.05 (*n* = 32)	Ineffective, *p* > 0.05 (*n* = 41)	Total ^a^ (*n* = 73)	
		*n* (%)	*n* (%)	*n* (%)	*p*=
**Commenced**					
**(Trimester)**					
	**1**	12 (41.4)	17 (58.6)	29	0.73
	**2**	18 (45.0)	22 (55.0)	40	0.83
	**3**	0 (0.0)	0 (0.0)	0	-
	**Unsure**	2 (50.0)	2 (50.0)	4	0.80
**Duration**					
**(Weeks)**					
	**<12**	2 (66.7)	1 (33.3)	3	0.42
	**12–29**	21 (40.4)	31 (59.6)	52	0.35
	**≥30**	7 (50.0)	7 (50.0)	14	0.61
	**Unsure**	2 (50.0)	2 (50.0)	4	0.80
**Intensity**					
**(Hours)**					
	**<4**	6 (33.3)	12 (66.7)	18	0.30
	**4–8**	3 (37.5)	5 (62.5)	8	0.70
	**>8**	14 (43.8)	18 (56.3)	32	0.99
	**Unsure**	9 (60.0)	6 (40.0)	15	0.16
**Frequency**					
**(Contact)**					
	**≤3**	4 (33.3)	8 (66.7)	12	0.42
	**4–7**	4 (30.8)	9 (69.2)	13	0.30
	**≥8**	19 (47.5)	21 (52.5)	40	0.49
	**Unsure**	5 (62.5)	3 (37.5)	8	0.26
**Delivery method**					
	**Individual**	14 (33.3)	28 (66.7)	42	0.04
	**Group ^b^**	15 (62.5)	9 (37.5)	24	0.02
	**Unsure**	3 (42.9)	4 (51.7)	7	0.96

^a^ No overall *p* value (or differing significance for sub-groups) and no control. ^b^ Combining interventions that involve group delivery and a combination of individual and group delivery.

## Supplementary Materials

The following are available online at http://www.mdpi.com/2072-6643/10/7/944/s1, Table S1: MEDLINE search strategy for interventions aimed at preventing excessive gestational weight gain (modified to suit other databases when necessary), Table S2: Description of diet interventions aimed at preventing excessive gestational weight gain, Table S3: The frequency of characteristics of all interventions aimed at preventing excessive gestational weight gain, Table S4: Description of physical activity interventions aimed at preventing excessive gestational weight gain, Table S5: Description of lifestyle interventions aimed at preventing excessive gestational weight gain, Table S6: Description of eHealth interventions aimed at preventing excessive gestational weight gain, Table S7: Description of “other” interventions aimed at preventing excessive gestational weight gain. Table S8: Meta-analyses (random-effect) of diet, physical activity, lifestyle and eHealth interventions. Studies with a high risk of bias removed. Figure S1: Meta-analysis (random-effect) of diet interventions aimed at preventing excessive gestational weight gain, Figure S2: Meta-analysis (random-effect) of physical activity interventions aimed at preventing excessive gestational weight gain, Figure S3: Meta-analysis (random-effect) of lifestyle interventions aimed at preventing excessive gestational weight gain, Figure S4: Meta-analysis (random-effect) of eHealth interventions aimed at preventing excessive gestational weight gain, Figure S5: Funnel-plot of meta-analysis (random-effect) of diet interventions aimed at preventing excessive gestational weight gain, Figure S6: Funnel-plot of meta-analysis (random-effect) of physical activity interventions aimed at preventing excessive gestational weight gain, Figure S7: Funnel-plot of meta-analysis (random-effect) of lifestyle interventions aimed at preventing excessive gestational weight gain, Figure S8: Funnel-plot of meta-analysis (random-effect) of eHealth interventions aimed at preventing excessive gestational weight gain.

## References

[1] Catalano P., De Mouzon S. (2015). Maternal obesity and metabolic risk to the offspring: Why lifestyle interventions may have not achieved the desired outcomes. Int. J. Obes..

[2] Rasmussen K., Abrams B., Bodnar L., Butte N., Catalano P., Siega-Riz A.M. (2010). Recommendations for weight gain during pregnancy in the context of the obesity epidemic. Obstet. Gynecol..

[3] Rasmussen K.M., Yaktine A.L., Institute of Medicine (US), National Research Council (US) (2009). Committee reexamine IOM pregnancy weight guidelines. Weight Gain during Pregnancy: Rexamining the Guidelines.

[4] De Jersey S., Nicholson J., Callaway L., Daniels L. (2012). A prospective study of pregnancy weight gain in Australian women. Aust. N. Z. J. Obstet. Gynaecol..

[5] Simas T., Liao X., Garrison A., Sullivan G., Howard A., Hardy J. (2011). Impact of updated Institute of Medicine guidelines on prepregnancy body mass index categorization, gestational weight gain recommendations, and needed counseling. J. Womens Health.

[6] Truong Y., Yee L., Caughey A., Cheng Y. (2015). Weight gain in pregnancy: Does the Institute of Medicine have it right?. Am. J. Obstet. Gynecol..

[7] Van Der Pligt P., Willcox J., Hesketh K., Ball K., Wilkinson S., Crawford D., Campbell K. (2013). Systematic review of lifestyle interventions to limit postpartum weight retention: Implications for future opportunities to prevent maternal overweight and obesity following childbirth. Obes. Rev..

[8] Paul K., Graham M., Olson C. (2013). The web of risk factors for excessive gestational weight gain in low income women. Matern. Child Health J..

[9] Schack-Nielsen L., Michaelsen K., Gamborg M., Mortensen E., Sørensen T. (2010). Gestational weight gain in relation to offspring body mass index and obesity from infancy through adulthood. Int. J. Obes..

[10] Siega-Riz A.M., Viswanathan M., Moos M., Deierlein A., Mumford S., Knaack J., Theida P., Lux L.J., Lohr K.N. (2009). A systematic review of outcomes of maternal weight gain according to the Institute of Medicine recommendations: Birthweight, fetal growth, and postpartum weight retention. Am. J. Obstet. Gynecol..

[11] Poston L. (2012). Gestational weight gain: Influences on the long-term health of the child. Curr. Opin. Clin. Nutr. Metab. Care.

[12] Hedderson M., Gunderson E., Ferrara A. (2010). Gestational weight gain and risk of gestational diabetes mellitus. Obstet. Gynecol..

[13] Fortner R., Pekow P., Solomon C., Markenson G., Chasan-Taber L. (2009). Prepregnancy body mass index, gestational weight gain, and risk of hypertensive pregnancy among Latina women. Am. J. Obstet. Gynecol..

[14] Stotland N., Hopkins L., Caughey A. (2004). Gestational weight gain, macrosomia, and risk of cesarean birth in nondiabetic nulliparas. Obstet. Gynecol..

[15] Goldstein R., Abell S., Ranasinha S., Misso M., Boyle J., Black M., Li N., Hu G., Corrado F., Rode L. (2017). Association of Gestational Weight Gain With Maternal and Infant Outcomes: A Systematic Review and Meta-analysis. JAMA.

[16] Olson C.M. (2008). Achieving a healthy weight gain during pregnancy. Annu. Rev. Nutr..

[17] Langley-Evans S. (2015). Nutrition in early life and the programming of adult disease: A review. J. Hum. Nutr. Diet..

[18] Brunner S., Stecher L., Ziebarth S., Nehring I., Rifas-Shiman S., Sommer C., Hauner H., von Kries R. (2015). Excessive gestational weight gain prior to glucose screening and the risk of gestational diabetes: A meta-analysis. Diabetologia.

[19] Tanentsapf I., Heitmann B., Adegboye A. (2011). Systematic review of clinical trials on dietary interventions to prevent excessive weight gain during pregnancy among normal weight, overweight and obese women. BMC Pregnancy Childbirth.

[20] Muktabhant B., Lumbiganon P., Ngamjarus C., Dowswell T. (2012). Interventions for preventing excessive weight gain during pregnancy. Cochrane Library.

[21] Streuling I., Beyerlein A., Von Kries R. (2010). Can gestational weight gain be modified by increasing physical activity and diet counseling? A meta-analysis of interventional trials. Am. J. Clin. Nutr..

[22] Thangaratinam S., Rogozińska E., Jolly K., Glinkowski S., Roseboom T., Tomlinson J., Kunz R., Mol B.W., Coomarasamy A., Khan K.S. (2012). Effects of interventions in pregnancy on maternal weight and obstetric outcomes: Meta-analysis of randomised evidence. BMJ.

[23] Dodd J., Grivell R., Crowther C., Robinson J. (2010). Antenatal interventions for overweight or obese pregnant women: A systematic review of randomised trials. BJOG.

[24] Skouteris H., Hartley-Clark L., McCabe M., Milgrom J., Kent B., Herring S., Gale J. (2010). Preventing excessive gestational weight gain: A systematic review of interventions. Obes. Rev..

[25] Campbell F., Johnson M., Messina J., Guillaume L., Goyder E. (2011). Behavioural interventions for weight management in pregnancy: A systematic review of quantitative and qualitative data. BMC Public Health.

[26] Haby K., Berg M., Gyllensten H., Hanas R., Premberg A. (2018). Mighty Mums—A lifestyle intervention at primary care level reduces gestational weight gain in women with obesity. BMC Obes..

[27] Yeo S., Samuel-Hodge C.D., Smith R., Leeman J., Ferraro A.M., Asafu-Adjei J.K. (2016). Challenges of integrating an evidence-based intervention in health departments to prevent excessive gestational weight gain among low-income women. Public Health Nurs..

[28] Eysenbach G. (2001). What is e-health?. J. Med. Internet Res..

[29] Syngelaki A., Nicolaides K., Balani J., Hyer S., Akolekar R., Kotecha R., Pastides A., Shehata H. (2016). Metformin versus Placebo in Obese Pregnant Women without Diabetes Mellitus. N. Engl. J. Med..

[30] Brownfoot F., Davey M., Kornman L., Brownfoot F. (2016). Routine weighing to reduce excessive antenatal weight gain: A randomised controlled trial. BJOG.

[31] Rogozinska E., Marlin N., Betran A., Astruup A., Barakat R., Bogaerts A., Cecatti J.C., Devlieger R., Dodd J.M., El Beltagy N. (2017). Effect of diet and physical activity based interventions in pregnancy on gestational weight gain and pregnancy outcomes: A meta-analysis of individual patient data from randomised trials. BMJ.

[32] Bennett C., Walker R., Blumfield M., Gwini S., Ma J., Wang F., Wan Y., Truby H. (2018). Interventions designed to reduce excessive gestational weight gain can reduce the incidence of gestational diabetes mellitus: A systematic review and meta-analysis of randomised controlled trials. Diabetes Res. Clin. Pract..

[33] Higgins J., Green S. (2011). Cochrane Handbook for Systematic Reviews of Interventions.

[34] Clapp J., Kim H., Burciu B., Schmidt S., Petry K., Lopez B. (2002). Continuing regular exercise during pregnancy: Effect of exercise volume on fetoplacental growth. Am. J. Obstet. Gynecol..

[35] Nobles C., Marcus B., Stanek E., Braun B., Whitcomb B., Manson J. (2018). The Effect of an Exercise Intervention on Gestational Weight Gain: The Behaviors Affecting Baby and You (B.A.B.Y.) Study: A Randomized Controlled Trial. Am. J. Health Promot..

[36] Markovic T., Muirhead R., Overs S., Ross G., Louie J., Kizirian N. (2016). Randomized Controlled Trial Investigating the Effects of a Low-Glycemic Index Diet on Pregnancy Outcomes in Women at High Risk of Gestational Diabetes Mellitus: The GI Baby 3 Study. Diabetes Care.

[37] Peccei A., Blake-Lamb T., Rahilly D., Hatoum I., Bryant A. (2017). Intensive prenatal nutrition counseling in a community health setting: A randomized controlled trial. Obstet. Gynecol..

[38] Rhodes E., Pawlak D., Takoudes T., Ebbeling C., Feldman H., Lovesky M., Cooke E.A., Leidig M.M., Ludwig D.S. (2010). Effects of a low-glycemic load diet in overweight and obese pregnant women: A pilot randomized controlled trial. Am. J. Clin. Nutr..

[39] Cahill A., Haire-Joshu D., Cade W., Stein R., Woolfolk C., Moley K., Mathur A., Schechtman K., Klein S. (2018). Weight control program and gestational weight gain in disadvantaged women with overweight or obesity: A randomized clinical trial. Obesity.

[40] Pawalia A., Kulandaivelan S., Savant S., Yadav V. (2017). Exercise in pregnancy: Effect on obesity parameters in indian women—A randomized controlled trial. Rom. J. Diabetes Nutr. Metab. Dis..

[41] Phelan S., Wing R., Brannen A., McHugh A., Hagobian T., Schaffner A., Jelalian E., Hart C.N., Scholl T.O., Munoz-Christian K. (2018). Randomized controlled clinical trial of behavioral lifestyle intervention with partial meal replacement to reduce excessive gestational weight gain. Am. J. Clin. Nutr..

[42] Simmons D., Jelsma J., Galjaard S., Devlieger R., van Assche A., Jans G., Corcoy R., Adelantado J.M., Dunne F., Desoye G. (2015). Results from a European multicenter randomized trial of physical activity and/or healthy eating to reduce the risk of gestational diabetes mellitus: The DALI lifestyle pilot. Diabetes Care.

[43] Thomson J., Tussing-Humphreys L., Goodman M., Olender S. (2016). Gestational weight gain: Results from the delta healthy sprouts comparative impact trial. J. Pregnancy.

[44] Moses R., Casey S., Quinn E., Cleary J., Tapsell L., Milosavljevic M., Petocz P., Brand-Miller J.C. (2014). Pregnancy and Glycemic Index Outcomes study: Effects of low glycemic index compared with conventional dietary advice on selected pregnancy outcomes. Am. J. Clin. Nutr..

[45] Perales M., Cordero Y., Vargas M., Lucia A., Barakat R. (2015). Exercise and depression in overweight and obese pregnant women: A randomised controlled trial. Arch. Med. Deporte.

[46] Abdel-Aziz S., Hegazy I., Mohamed D., Abu El Kasem M., Hagag S. (2018). Effect of dietary counseling on preventing excessive weight gain during pregnancy. Public Health.

[47] Vítolo M., Bueno M., Gama C. (2011). Impact of a dietary counseling program on the gain weight speed of pregnant women attended in a primary care service. Rev. Bras. Ginecol. Obstet..

[48] Ramírez-Vélez R., Lobelo F., Aguilar-de Plata A., Izquierdo M., García-Hermoso A. (2017). Exercise during pregnancy on maternal lipids: A secondary analysis of randomized controlled trial. BMC Pregnancy Childbirth.

[49] Renault K., Nørgaard K., Nilas L., Carlsen E., Cortes D., Pryds O., Secher N.J. (2014). The Treatment of Obese Pregnant Women (TOP) study: A randomized controlled trial of the effect of physical activity intervention assessed by pedometer with or without dietary intervention in obese pregnant women. Am. J. Obstet. Gynecol..

[50] Wang S., Ma J., Yang H. (2015). Lifestyle intervention for gestational diabetes mellitus prevention: A cluster-randomized controlled study. Chronic Dis. Transl. Med..

[51] Bosaeus M., Hussain A., Karlsson T., Andersson L., Hulthen L., Svelander C., Sandberg A.S., Larsson I., Ellegard L., Holmang A. (2015). A randomized longitudinal dietary intervention study during pregnancy: Effects on fish intake, phospholipids, and body composition. Nutrition.

[52] Vinter C., Jensen D., Ovesen P., Beck-Nielsen H.J.O. (2011). Lifestyle and pregnancy (LIP) study: The clinical effect of lifestyle intervention during pregnancy in obese women. Diabetes.

[53] Sterne J. (2009). Meta-Analysis in Stata: An Updated Collection from the Stata Journal.

[54] Egger M., Smith G., Schneider M., Minder C. (1997). Bias in meta-analysis detected by a simple, graphical test. BMJ.

[55] Borenstein M. (2009). Introduction to meta-analysis.

[56] Racey M., O’Brien C., Douglas S., Marquez O., Hendrie G., Newton G. (2016). Systematic review of school-based interventions to modify dietary behavior: Does intervention intensity impact effectiveness?. J. Sch. Health.

[57] Hendrie G.A., Brindal E., Baird D., Gardner C. (2013). Improving children’s dairy food and calcium intake: Can intervention work? A systematic review of the literature. Public Health Nutr..

[58] Hingle M.D., O’Connor T.M., Dave J.M., Baranowski T. (2010). Parental involvement in interventions to improve child dietary intake: A systematic review. Prev. Med..

[59] StataCorp (2011). Stata Statistical Software: Release 12.

[60] Korpi-Hyovalti E., Schwab U., Laaksonen D.E., Linjama H., Heinonen S., Niskanen L. (2012). Effect of intensive counselling on the quality of dietary fats in pregnant women at high risk of gestational diabetes mellitus. Br. J. Nutr..

[61] McCarthy E., Walker S., Ugoni A., Lappas M., Leong O., Shub A. (2016). Self-weighing and simple dietary advice for overweight and obese pregnant women to reduce obstetric complications without impact on quality of life: A randomised controlled trial. BJOG.

[62] Walsh J., McGowan C., Mahony R., Foley M., McAuliffe F. (2012). Low glycaemic index diet in pregnancy to prevent macrosomia (ROLO study): Randomised control trial. BMJ.

[63] Thornton Y., Smarkola C., Kopacz S., Ishoof S. (2009). Perinatal outcomes in nutritionally monitored obese pregnant women: A randomized clinical trial. J. Natl. Med. Assoc..

[64] Deveer R., Deveer M., Akbaba E., Engin-Üstün Y., Aydoǧan P., Çelikkaya H., Danisman N., Mollamahmutoglu L. (2013). The effect of diet on pregnancy outcomes among pregnants with abnormal glucose challenge test. Eur. Rev. Med. Pharmacol. Sci..

[65] Di Carlo C., Iannotti G., Sparice S., Chiacchio M., Greco E., Tommaselli G., Nappi C. (2014). The role of a personalized dietary intervention in managing gestational weight gain: A prospective, controlled study in a low-risk antenatal population. Arch. Gynecol. Obstet..

[66] Bonomo M., Corica D., Mion E., Goncalves D., Motta G., Merati R., Ragusa A., Morabito A. (2005). Evaluating the therapeutic approach in pregnancies complicated by borderline glucose intolerance: A randomized clinical trial. Diabet. Med..

[67] Wolff S., Legarth J., Vangsgaard K., Toubro S., Astrup A. (2008). A randomized trial of the effects of dietary counseling on gestational weight gain and glucose metabolism in obese pregnant women. Int. J. Obes..

[68] Ilmonen J., Isolauri E., Poussa T., Laitinen K. (2011). Impact of dietary counselling and probiotic intervention on maternal anthropometric measurements during and after pregnancy: A randomized placebo-controlled trial. Clin. Nutr..

[69] Barakat R., Cordero Y., Coteron J., Luaces M., Montejo R. (2012). Exercise during pregnancy improves maternal glucose screen at 24–28 weeks: A randomised controlled trial. Br. J. Sports Med..

[70] Barakat R., Lucia A., Ruiz J. (2009). Resistance exercise training during pregnancy and newborn’s birth size: A randomised controlled trial. Int. J. Obes..

[71] Barakat R., Pelaez M., Cordero Y., Perales M., Lopez C., Coteron J., Mottola M.F. (2016). Exercise during pregnancy protects against hypertension and macrosomia: Randomized clinical trial. Am. J. Obstet. Gynecol..

[72] Barakat R., Pelaez M., Lopez C., Lucia A., Ruiz J.R. (2013). Exercise during pregnancy and gestational diabetes-related adverse effects: A randomised controlled trial. Br. J. Sports Med..

[73] Barakat R., Pelaez M., Montejo R., Luaces M., Zakynthinaki M. (2011). Exercise during pregnancy improves maternal health perception: A randomized controlled trial. Am. J. Obstet. Gynecol..

[74] Barakat R., Pelaez M., Montejo R., Refoyo I., Coteron J. (2014). Exercise throughout pregnancy does not cause preterm delivery: A randomized, controlled trial. J. Phys. Act. Health.

[75] Ruiz J., Perales M., Pelaez M., Lopez C., Lucia A., Barakat R. (2013). Supervised exercise-based intervention to prevent excessive gestational weight gain: A randomized controlled trial. Mayo Clin. Proc..

[76] Clapp J., Kim H., Burciu B., Lopez B. (2000). Beginning regular exercise in early pregnancy: Effect on fetoplacental growth. Am. J. Obstet. Gynecol..

[77] Garshasbi A., Faghih Zadeh S. (2005). The effect of exercise on the intensity of low back pain in pregnant women. Int. J. Gynecol. Obstet..

[78] Cavalcante S., Cecatti J., Pereira R., Baciuk E., Bernardo A., Silveira C. (2009). Water aerobics II: Maternal body composition and perinatal outcomes after a program for low risk pregnant women. Reprod. Health.

[79] Haakstad L., Bø K. (2011). Effect of regular exercise on prevention of excessive weight gain in pregnancy: A randomised controlled trial. Eur. J. Contracept. Reprod. Health Care.

[80] Nascimento S., Surita F., Parpinelli M., Siani S., Pinto e Silva J. (2011). The effect of an antenatal physical exercise programme on maternal/perinatal outcomes and quality of life in overweight and obese pregnant women: A randomised clinical trial. BJOG.

[81] Kong K., Campbell C., Foster R., Peterson A., Lanningham-Foster L. (2014). A pilot walking program promotes moderate-intensity physical activity during pregnancy. Med. Sci. Sports Exerc..

[82] Bisson M., Almeras N., Dufresne S., Robitaille J., Rheaume C., Bujold E., Frenette J., Tremblay A., Marc I. (2015). A 12-week exercise program for pregnant women with obesity to improve physical activity levels: An open randomised preliminary study. PLoS ONE.

[83] Ronnberg A., Ostlund I., Fadl H., Gottvall T., Nilsson K. (2015). Intervention during pregnancy to reduce excessive gestational weight gain-a randomised controlled trial. BJOG.

[84] Dekker Nitert M., Barrett H., Denny K., McIntyre H., Callaway L. (2015). Exercise in pregnancy does not alter gestational weight gain, MCP-1 or leptin in obese women. Aust. N. Z. J. Obstet. Gynaecol..

[85] Oostdam N., Van Poppel M., Wouters M., Eekhoff E., Bekedam D., Kuchenbecker W., Quartero H., Heres M., van Mechelen W. (2012). No effect of the FitFor2 exercise programme on blood glucose, insulin sensitivity, and birthweight in pregnant women who were overweight and at risk for gestational diabetes: Results of a randomised controlled trial. BJOG.

[86] Bacchi M., Mottola M., Perales M., Refoyo I., Barakat R. (2018). Aquatic activities during pregnancy prevent excessive maternal weight gain and preserve birth weight: A randomized clinical trial. Am. J. Health Promot..

[87] Barakat R., Franco E., Perales M., Lopez C., Mottola M. (2018). Exercise during pregnancy is associated with shorter duration of labor: A randomized clinical trial. Eur. J. Obstet. Gynecol. Reprod. Biol..

[88] da Silva S., Hallal P., Domingues M., Bertoldi A., Silveira M., Bassani D., da Silva I.C., da Sliva B.C., Coll C.D., Evenson K. (2017). A randomized controlled trial of exercise during pregnancy on maternal and neonatal outcomes: Results from the PAMELA study. Int. J. Behav. Nutr. Phys. Act..

[89] Daly N., Farren M., McKeating A., O’Kelly R., Stapleton M., Turner M. (2017). A medically supervised pregnancy exercise intervention in obese women: A randomized controlled trial. Obstet. Gynecol..

[90] Garnæs K., Mørkved S., Salvesen Ø., Moholdt T. (2016). Exercise training and weight gain in obese pregnant women: A randomized controlled trial (ETIP Trial). PLoS Med..

[91] Wang C., Wei Y., Zhang X., Zhang Y., Xu Q., Sun Y., Su S., Zhang L., Liu C., Feng Y. (2017). A randomized clinical trial of exercise during pregnancy to prevent gestational diabetes mellitus and improve pregnancy outcome in overweight and obese pregnant women. Am. J. Obstet. Gynecol..

[92] Rodríguez-Blanque R., Carlos Sánchez-García J., Manuel Sánchez-López A., Mur-Villar N., Fernández-Castillo R., José Aguilar-Cordero M. (2017). Influence of physical exercise during pregnancy on birthweight: A randomized clinical trial. Nutr. Hosp..

[93] Petrella E., Malavolti M., Bertarini V., Pignatti L., Neri I., Battistini N.C., Facchinetti F. (2014). Gestational weight gain in overweight and obese women enrolled in a healthy lifestyle and eating habits program. J. Matern. Neonatal Med..

[94] Phelan S., Phipps M., Abrams B., Darroch F., Schaffner A., Wing R. (2011). Randomized trial of a behavioral intervention to prevent excessive gestational weight gain: The Fit for Delivery Study. Am. J. Clin. Nutr..

[95] Polley B., Wing R., Sims C. (2002). Randomized controlled trial to prevent excessive weight gain in pregnant women. Int. J. Obes..

[96] Poston L., Bell R., Croker H., Flynn A., Godfrey K., Goff L., Hayes L., Khazaezadeh N., Nelson S.M., Oteng-Ntim E. (2015). Effect of a behavioural intervention in obese pregnant women (the UPBEAT study): A multicentre, randomised controlled trial. Lancet Diabetes Endocrinol..

[97] Asbee S., Jenkins T., Butler J., White J., Elliot M., Rutledge A. (2009). Preventing excessive weight gain during pregnancy through dietary and lifestyle counseling: A randomized controlled trial. Obstet. Gynecol..

[98] Aşcı Ö., Rathfisch G. (2016). Effect of lifestyle interventions of pregnant women on their dietary habits, lifestyle behaviors, and weight gain: A randomized controlled trial. J. Health Popul. Nutr..

[99] Guelinckx I., Devlieger R., Mullie P., Vansant G. (2010). Effect of lifestyle intervention on dietary habits, physical activity, and gestational weight gain in obese pregnant women: A randomized controlled trial. Am. J. Clin. Nutr..

[100] Huang T., Yeh C., Tsai Y. (2011). A diet and physical activity intervention for preventing weight retention among Taiwanese childbearing women: A randomised controlled trial. Midwifery.

[101] Hui A., Back L., Ludwig S., Gardiner P., Sevenhuysen G., Dean H., Sellers E., McGavock J., Morris M., Bruce S. (2012). Lifestyle intervention on diet and exercise reduced excessive gestational weight gain in pregnant women under a randomized controlled trial. Obstet. Gynecol. Surv..

[102] Hui A., Back L., Ludwig S., Gardiner P., Sevenhuysen G., Dean H., Sellers E., McGavock J., Morris M., Jiang D. (2014). Effects of lifestyle intervention on dietary intake, physical activity level, and gestational weight gain in pregnant women with different pre-pregnancy Body Mass Index in a randomized control trial. BMC Pregnancy Childbirth.

[103] Ruchat S., Davenport M., Giroux I., Hillier M., Batada A., Sopper M., Hammond J.M., Mottola M.F. (2012). Nutrition and exercise reduce excessive weight gain in normal-weight pregnant women. Med. Sci. Sports Exerc..

[104] Bogaerts A., Devlieger R., Nuyts E., Witters I., Gyselaers W., Van den Bergh B. (2013). Effects of lifestyle intervention in obese pregnant women on gestational weight gain and mental health: A randomized controlled trial. Int. J. Obes..

[105] Harrison C., Lombard C., Strauss B., Teede H. (2013). Optimizing healthy gestational weight gain in women at high risk of gestational diabetes: A randomized controlled trial. Obesity.

[106] Hawkins M., Hosker M., Marcus B., Rosal M., Braun B., Stanek E., Markenson G., Chasan-Taber L. (2015). A pregnancy lifestyle intervention to prevent gestational diabetes risk factors in overweight Hispanic women: A feasibility randomized controlled trial. Diabet. Med..

[107] Rauh K., Gabriel E., Kerschbaum E., Schuster T., von Kries R., Amann-Gassner U., Hauner H. (2013). Safety and efficacy of a lifestyle intervention for pregnant women to prevent excessive maternal weight gain: A cluster-randomized controlled trial. BMC Pregnancy Childbirth.

[108] Gesell S., Katula J., Strickland C., Vitolins M. (2015). Feasibility and initial efficacy evaluation of a community-based cognitive-behavioral lifestyle intervention to prevent excessive weight gain during pregnancy in Latina women. Matern. Child Health J..

[109] Skouteris H., McPhie S., Hill B., McCabe M., Milgrom J., Kent B., Bruce L., Herring S., Gale J., Mihalopoulos C. (2016). Health coaching to prevent excessive gestational weight gain: A randomized-controlled trial. Br. J. Health Psychol..

[110] Sagedal L., Øverby N., Bere E., Torstveit M., Lohne-Seiler H., Småstuen M., Hillesund E.R., Henriksen T., Vistad I. (2016). Lifestyle intervention to limit gestational weight gain: The Norwegian Fit for Delivery randomised controlled trial. BJOG.

[111] Althuizen E., van der Wijden C., van Mechelen W., Seidell J., van Poppel M. (2013). The effect of a counselling intervention on weight changes during and after pregnancy: A randomised trial. BJOG.

[112] Dodd J., Turnbull D., McPhee A., Deussen A., Grivell R., Yelland L., Crowther C.A., Wittert G., Owens J.A., Robinson J.S. (2014). Antenatal lifestyle advice for women who are overweight or obese: LIMIT randomised trial. BMJ.

[113] Vesco K., Karanja N., King J., Gillman M., Leo M., Perrin N., McEvoy C.T., Eckhardt C.L., Smith K.S., Stevens C.L. (2014). Efficacy of a group-based dietary intervention for limiting gestational weight gain among obese women: A randomized trial. Obesity.

[114] Jing W., Huang Y., Liu X., Luo B., Yang Y., Liao S. (2015). The effect of a personalized intervention on weight gain and physical activity among pregnant women in China. Int. J. Gynaecol. Obstet..

[115] Luoto R., Kinnunen T., Aittasalo M., Ojala K., Mansikkamaki K., Toropainen E., Kolu P., Vasankari T. (2010). Prevention of gestational diabetes: Design of a cluster-randomized controlled trial and one-year follow-up. BMC Pregnancy Childbirth.

[116] Bruno R., Petrella E., Bertarini V., Pedrielli G., Neri I., Facchinetti F. (2017). Adherence to a lifestyle programme in overweight/obese pregnant women and effect on gestational diabetes mellitus: A randomized controlled trial. Matern. Child Nutr..

[117] Herring Cruice J., Bennett G., Rose M., Davey A., Foster G. (2016). Preventing excessive gestational weight gain among African American women: A randomized clinical trial. Obesity.

[118] Willcox J., Wilkinson S., Lappas M., Ball K., Crawford D., McCarthy E., Fjeldsoe B., Whittaker R., Maddison R., Campbell K. (2017). A mobile health intervention promoting healthy gestational weight gain for women entering pregnancy at a high body mass index: The txt4two pilot randomised controlled trial. BJOG.

[119] Olsen C., Groth S., Graham M., Reschke J., Strawderman M., Fernandez D. (2018). The effectiveness of an online intervention in preventing excessive gestational weight gain: The e-moms roc randomized controlled trial. BMC Pregnancy Childbirth.

[120] Smith K., Lanningham-Foster L., Welch A., Campbell C. (2016). Web-Based Behavioral Intervention Increases Maternal Exercise but Does Not Prevent Excessive Gestational Weight Gain in Previously Sedentary Women. J. Phys. Act. Health.

[121] Jackson R., Stotland N., Caughey A., Gerbert B. (2011). Improving diet and exercise in pregnancy with Video Doctor counseling: A randomized trial. Patient Educ. Couns..

[122] Daley A., Jolly K., Jebb S., Lewis A., Clifford S., Roalfe A., Kenyon S., Aveyard P. (2015). Feasibility and acceptability of regular weighing, setting weight gain limits and providing feedback by community midwives to prevent excess weight gain during pregnancy: Randomised controlled trial and qualitative study. BMC Obes..

[123] Jeffries K., Shub A., Walker S., Hiscock R., Permezel M. (2009). Reducing excessive weight gain in pregnancy: A randomised controlled trial. Med. J. Aust..

[124] Quinlivan J., Lam L., Fisher J. (2011). A randomised trial of a four-step multidisciplinary approach to the antenatal care of obese pregnant women. Aust. N. Z. J. Obstet. Gynaecol..

[125] Santamaria A., Di Benedetto A., Petrella E., Pintaudi B., Corrado F., D’Anna R., Neri I., Facchinetti F. (2016). Myo-inositol may prevent gestational diabetes onset in overweight women: A randomized, controlled trial. J. Matern. Fetal Neonatal Med..

[126] Herrera-Perdigon J., Hopkins E., Marcalle M., Brooten D., Youngblut J., Lizardo M. (2005). Weight gain in high-risk pregnant women: Comparison by primary diagnosis and type of care. Clin. Excell. Nurse Pract..

[127] Bechtel-Blackwell D. (2002). Computer-assisted self-interview and nutrition education in pregnant teens. Clin. Nurs. Res..

[128] Sterne J., Sutton A., Ioannidis J., Terrin N., Jones D., Lau J., Carpenter J., Rucker G., Harbord R.M., Schmid C.H. (2011). Recommendations for examining and interpreting funnel plot asymmetry in meta-analyses of randomised controlled trials. BMJ.

[129] Willimas R., Wood L., Collins C., Callister R. (2015). Effectiveness of weight loss interventions—Is there a difference between men and women: A systematic review. Obes. Rev..

[130] Knight M., Foster C. (2017). Diet and exercise in pregnancy. BMJ.

[131] Di Cesare M., Bentham J., Stevens G., Zhou B., Danaei G., Lu Y., Bixby H., Cowan M.J., Riley L.M., Hajifathalian K. (2016). Trends in adult body-mass-index in 200 countries from 1975 to 2014: A pooled analysis of 1698 population-based measurement studies with 19.2 million participants. Lancet.

[132] Heslehurst N., Rankin J., Wilkinson J., Summerbell C. (2010). A nationally representative study of maternal obesity in England, UK: Trends in incidence and demographic inequalities in 619,323 births, 1989–2007. Int. J. Obes..

[133] Hebden L., Chey T., Allman-Farinelli M. (2012). Lifestyle intervention for preventing weight gain in young adults: A systematic review and meta-analysis of RCTs. Obes. Rev..

[134] Chi Y., Sha F., Yip P., Chen J., Chen Y. (2016). Randomized comparison of group versus individual educational interventions for pregnant women to reduce their secondhand smoke exposure. Medicine.

[135] Lombard C., Deeks A., Jolley D., Ball K., Teede H. (2010). A low intensity, community based lifestyle programme to prevent weight gain in women with young children: Cluster randomised controlled trial. BMJ.

[136] Lombard C., Harrison C., Kozica S., Zoungas S., Keating C., Teede H. (2014). Effectiveness and implementation of an obesity prevention intervention: The HeLP-her Rural cluster randomised controlled trial. BMC Public Health.

[137] Gilmore L., Klempel-Donchenko M., Redman L. (2015). Pregnancy as a window to future health: Excessive gestational weight gain and obesity. Semin. Perinatol..

[138] Tomedi L., Simhan H., Chang C., McTigue K., Bodnar L. (2014). Gestational weight gain, early pregnancy maternal adiposity distribution, and maternal hyperglycemia. Matern. Child Health J..

[139] Walker R., Kumar A., Blumfield M., Truby H. (2018). Maternal nutrition and weight management in pregnancy: A nudge in the right direction. Nutr. Bull..

[140] Stephenson J., Heslehurst N., Hall J., Schoenaker D., Hutchison J., Cade J., Poston L., Barrett G., Crozier S.R., Barker M. (2018). Before the beginning: Nutrition and lifestyle in the preconception period and its importance for future health. Lancet.

[141] Centers of Disease Control and Prevention (CDC) Antropometric Reference Data for Children and Adults: United States, 2007–2010. https://www.cdc.gov/nchs/data/series/sr_11/sr11_252.pdf.

[142] Steer P. (2015). Routine weighing of women during pregnancy is of limited value and should be abandoned: FOR: Routine weighing does not solve the problem of obesity in pregnancy. BJOG.

[143] Dawes M., Grudzinskas J. (1991). Repeated measurement of maternal weight during pregnancy. Is this a useful practice?. BJOG.

[144] Allen-Walker V., Woodside J., Holmes V., Young I., Cupples M.E., Hunter A., McKinley M.C. (2016). Routine weighing of women during pregnancy-is it time to change current practice?. BJOG.

[145] Brown A., Avery A. (2012). Healthy weight management during pregnancy: What advice and information is being provided. J. Hum. Nutr. Dietetics.

[146] Schmied V., Duff M., Dahlen H., Mills A., Kolt G. (2011). ‘Not waving but drowning’: A study of the experiences and concerns of midwives and other health professionals caring for obese childbearing women. Midwifery.

[147] Stotland N.E., Gilbert P., Bogetz A., Harper C.C., Abrams B., Gerbert B. (2010). Preventing excessive weight gain in pregnancy: How do prenatal care providers approach counseling?. J. Womens Health.

[148] Walker R., Mazza D., Blumfield M., Bennett C., Truby H. (2017). Maternal gestational weight gain during pregnancy: Prioritising the conversation. Aust. J. Prim. Health.

[149] Herring S. (2017). Do mHealth interventions prevent excessive gestational weight gain?. BJOG.

[150] National Institute for Health and Care Excellence (NICE) Dietary Interventions and Advice for Adults. https://pathways.nice.org.uk/pathways/diet/dietary-interventions-and-advice-for-adults#coentent=view-node%3Anodes-advice-for-all-women.

[151] National Health and Medical Research Council (NHMRC) Clinical Practice Guidelines for the Management of Overweight and Obesity in Adults, Adolescents and Children in Australia. https://www.nhmrc.gov.au/_files_nhmrc/publications/attachments/n57_obesity_guidelines_140630.pdf.

[152] Higgins J., Thompson G., Deeks J., Altman D. (2003). Measuring inconsistency in meta-analyses. BMJ.

[153] Krukowski R., West D., Dicarlo M., Shankar K., Cleves M., Saylors M., Andrea A. (2016). Are early first trimester weights valid proxies for preconception weight?. BMC Pregnancy Childbirth.

[154] Jaacks L., Kavle J., Perry A., Myaku A. (2017). Programming maternal and child overweight and obesity in the context of undernutrition: Current evidence and key considerations for low and middle-income countries. Public Health Nutr..

