# Supplementary material for: Attenuating Pregnancy Weight Gain—What Works and Why: A Systematic Review and Meta-Analysis
Source: Nutrients. 2018 Jul 22;10(7):944. doi: 10.3390/nu10070944 (PMC6073617; doi:10.3390/nu10070944)
Supplement: Supplementary file 1 [file nutrients-10-00944-s001.zip › Table S1_Medline Search Strategy.pdf]

Table S1: MEDLINE search strategy for interventions aimed at preventing excessive gestational weight gain (modified to suit other databases when necessary)

1	pregnan*.tw
2	exp Pregnancy/
3	gestational diabetes.tw
4	exp Diabetes, Gestational/
5	antenatal.tw
6	exp Prenatal Care/
7	1 or 2 or 3 or 4 or 5 or 6
8	intervention*.tw
9	Exp Early Medical Intervention/
10	diet*.tw
11	nutrition*.tw
12	exp Diet/ or exp Diet Therapy/
13	pharmacotherapy*.tw
14	exp Drug Therapy/
15	(eHealth or e-Health or mHealth or m-Health).tw

16	(smartphone or SMS or text messag*).tw
17	Exp Text Messaging/
18	Exp Telemedicine/
19	exercise*.tw
20	(physic* adj1 activ*).tw
21	Exp Exercise/ or exp Exercise Therapy/
22	sleep*.tw
23	(nap or naps or napping).tw
24	snor*.te
25	apn?ea.tw
26	exp Sleep/
27	counsel*.tw
28	exp Counseling/
29	exp Behavior Therapy
30	lifestyle.tw
31	life style.tw
32	exp Life Style/

33	8 or 9 or 10 or 11 or 12 or 13 or 14 or 15 or 16 or 17 or 18 or 19 or 20 or 21 or 22 or 23 or 24 or 25 or 26 or 27 or 28 or 29 or 30 or 31 or 32
34	weight.tw
35	exp body weight/ or exp birth weight/ or exp body weight changes/ or exp weight gain/ or exp weight loss/
36	34 or 35
37	7 and 33 and 36
38	Randomi#ed controlled trial.pt
29	Controlled clinical trial.pt
40	randomi#ed.ab.
41	placebo.ab
42	clinical trials as topic.sh.
43	randomly.ab
44	trial.ti
45	38 or 39 or 40 or 41 or 42 or 43 or 44
46	Animals/
47	Humans/

48	46 not 47
49	45 not 48
50	37 and 49
