# Supplementary material for: Attenuating Pregnancy Weight Gain—What Works and Why: A Systematic Review and Meta-Analysis
Source: Nutrients. 2018 Jul 22;10(7):944. doi: 10.3390/nu10070944 (PMC6073617; doi:10.3390/nu10070944)
Supplement: Supplementary file 1 [file nutrients-10-00944-s001.zip › Table S2_ Diet Interventions_Results Summary.pdf]

Table S2: Description of diet interventions aimed at preventing excessive gestational weight gain.

First author	Setting and date	Population	Intervention	GWG[a] and GWG measure	Quality[b]
Abdel-Aziz[46]	Cairo, Egypt<br>Jul 2015 –<br>Jan 2017	Primigravidae, 20-30 years <12 weeks gestation.<br>Excluded: <18 years, history of stillbirth, abortion, chronic disease, medications that interfere with weight.<br>Sample: 147<br>Final analyses: 75 int, 72 con	Tailored nutritional counselling sessions with counsellor every 2 weeks plus 3 brief phone calls.<br>Duration: 12 weeks (intervention phase)	Int: 42.7%[c]<br>Con: 13.9%[c]<br>$p < 0.01$<br>GWG: First – last antenatal visit	Random: Low<br>Allocation: Low<br>Blinding: Unclear<br>Data: Unclear<br>SR: Unclear<br>Other: Unclear
Bonomo[66]	Milan, Italy<br>1997 – 2002	Singleton pregnancy, elevated GCT (>7.8mmol/L), Caucasian.<br>Sample: 450 (150 int, 150 SM, 150 con)<br>Final analyses: 150 int, 150 SM, 150 con	Int: Dietary advice based on pre-pregnancy weight (24-30kcal/kg/day). Outpatient clinic every 2 weeks.<br>SM: No special care, diet or pharmacological treatment.<br>Duration: Unclear	Int: 13.1±4.3<br>SM: 12.6±3.9<br>Con: 13.6±4.4<br>*Not significant*<br>GWG: Unclear	Random: Low<br>AC: Unclear<br>Blinding: Unclear<br>Data: Unclear<br>SR: Unclear<br>Other: Low
Bosaeus[51]	Gothenburg, Sweden<br>Apr 2009 –<br>Dec 2012	Singleton pregnancy, 20-45 years, healthy weight.<br>Excluded: Non-European, diabetes, use of neuroleptic drugs, vegetarían, vegan.<br>Sample: 101 (49 int, 52 con)<br>Final analyses: 18 int, 17 con	One counselling session per trimester and 5 phone calls. Women advised to: consume 3 meals of fish each week, decrease sugar intake <10% energy, consume >500g vegetables and fruit each day, increase energy by 350kcal/day in 2nd trimester and 500kcal/day in 3rd trimester.<br>Duration: 28-32 weeks	Int:11.6 (8.9-13.9)[d]<br>Con: 11.6 (9.5-11.6)[d]<br>$p = 0.96$<br>GWG: 1st - 3rd trimester	Random: Low<br>AC: Unclear<br>Blinding: Unclear<br>Data: High<br>SR: Unclear<br>Other: Unclear
Deveer[64]	Turkey	Singleton pregnancy, positive 50g GCT and negative 100g OGTT between 24-28 weeks gestation.<br>Excluded: diabetes, history of still birth, active chronic systemic disease.<br>Sample: 100 (50 int, 50 con)<br>Final analyses: 50 int, 50 con	Individualized dietary advice (1800-2500kcal/day). Follow up weekly in the first month and then every 2 weeks until delivery.<br>Duration: 12-16 weeks	Int: 12.6±3.9<br>Con: 16.1±4.1<br>$p < 0.01$<br>GWG: Pre-pregnancy - delivery	Random: High<br>AC: Unclear<br>Blinding: Unclear<br>Data: Low<br>SR: Unclear<br>Other: Unclear

Di Carlo[65]	Naples, Italy<br>Jun 2010 –<br>Jun 2011	Singleton pregnancy, < 12 weeks gestation.<br>Excluded: HTN, thyroid disorder, BMI <20 or >40.<br>Sample: 154 (77 int, 77 con)<br>Final analyses: 61 int, 59 con	A personalised meal plan (average intake 1916kcal/day) and monthly reviews with a dietitian.<br>Duration: 32 weeks	Int: 8.2±4.0<br>Con: 13.4±4.2<br>$p < 0.01$<br>GWG: Pre-pregnancy - delivery	Random: Low<br>AC: Low<br>Blinding: Unclear<br>Data: Low<br>SR: Unclear<br>Other: Low
Ilmonen[68]	Finland<br>Apr 2002-<br>Nov 2005	Singleton pregnancy, <17 weeks gestation, no metabolic diseases.<br>Sample: 256 (85 diet/probiotic, 86 diet/placebo, 85 con/placebo)<br>Final analyses: 64 diet/probiotic, 64 diet/placebo, 57 con/placebo	Diet: Recommended energy 55- 60% CHO, 10-15% protein, 30% fat.<br>Probiotic: 1 capsule per day of *L.rhamnosus* GG and *B.lactus*<br>Duration: 30 weeks	Diet/probiotic: 14.8±4.4<br>Diet/placebo: 14.7±5.0<br>Diet/Con: 18.8±5.2<br>$p = 0.98$<br>GWG: Pre-pregnancy - delivery	Random: Low<br>AC: Low<br>Blind: Low<br>Data: Unclear<br>SR: Unclear<br>Other: Unclear
Korpi-Hyovalti[60]	Finland	Singleton pregnancy, 8-12 weeks gestation, risk of GDM.<br>Excluded: GDM.<br>Sample: 60 (30 int, 30 con)<br>Final analyses: 27 int, 27 con	10 individual dietary counselling sessions throughout pregnancy. Recommended energy 50-55% CHO, 15-20% protein, 30% fat<br>Duration: 28-32 weeks	Int: 11.4±6.0<br>Con: 13.9±5.1<br>$p = 0.06$<br>GWG: Pre-pregnancy - delivery	Random: Low<br>AC: Unclear<br>Blinding: Unclear<br>Data: Low<br>SR: Unclear<br>Other: Low
Markovic[36]	Camperdown, Australia<br>Jan 2011 -<br>Oct 2012	Singleton pregnancy, 12-20 weeks gestation, high risk of GDM<br>Excluded: Pre-existing diabetes, special dietary requirements.<br>Sample: 147 (76 low-GI, 71 HF)<br>Final analyses: 65 low-GI, 60 HF	5 individual dietary consultations with a dietitian. Recommended energy 40-45% CHO, 15-25% protein, 25-30% fat.<br>Low-GI: Target GI ≤50<br>HF: Target GI 60<br>Duration: 20-26 weeks	Completers data<br>Low-GI: 11.0±5.3<br>HF: 10.7±5.7<br>$p = 0.79$<br>GWG: Unclear	Random: Low<br>AC: Low<br>Blinding: Low<br>Data: Low<br>SR: Unclear<br>Other: Low
McCarthy[61]	Melbourne, Australia<br>Apr 2011 –<br>Dec 2011	Singleton pregnancy, <20 weeks gestation , >18 years old, overweight or obese.<br>Excluded: Diabetes.<br>Sample: 382 (190 int, 192 con)<br>Final analyses: 159 int, 154 con	A counselling session with simple dietary advice based on the Australian Guide to Healthy Eating. Self-weighing encouraged.<br>Duration: 25 weeks	Overweight Int: 9.9±4.4<br>Overweight Con: 10.5±4.2<br>$p = 0.37$<br>Obese Int: 7.4±6.1<br>Obese Con: 8.8±6.6<br>$p = 0.17$<br>GWG: Pre-pregnancy – 36 weeks	Random: Low<br>AC: Low<br>Blinding: Unclear<br>Data: Low<br>SR: Unclear<br>Other: Low
Moses[44]	Australia<br>Feb 2010 –<br>Sep 2012	Singleton pregnancy, ≥18 years old, < 20 weeks gestation.<br>Excluded: Diabetes, special dietary requirements, medical conditions that may compromise metabolic status, medications known to affect body weight.<br>Sample: 691 (337 healthy eating, 354 low-GI)	Healthy eating: Advice based on the Australian Guide to Healthy Eating<br>Low-GI: Advice based on low-GI diet.<br>4 counselling sessions for both groups (3 face-to-face and a phone call)<br>Duration: 18 weeks	Low-GI: 10.2±3.3<br>Healthy eating: 10.3±5.1<br>$p = 0.73$<br>GWG: Randomization - 34 weeks	Random: Unclear<br>AC: Unclear<br>Blinding: Unclear<br>Data: Low<br>SR: Unclear<br>Other: Low

		Final analyses: 296 healthy eating, 280 low-GI			
Peccei[37]	United States<br>Dec 2009 – Aug 2014	Singleton pregnancy, <16 weeks, 18-49 years, BMI >25, <40.<br>Excluded: History of eating disorder<br>Sample: 300 (200 int, 100 con)<br>Final analysis: 168 int, 87 con	Both groups received face-to-face counselling, brochure, booklet at initial visit. Intervention group also received bi-weekly individualised sessions, meal plans and weight gain tracker.<br>Duration: 24 weeks	Int: 11.0±0.41<br>Con: 12.2±0.7<br>*Significant*<br>GWG: Weight at first visit – weight at last visit	Random: Low<br>AC: Unclear<br>Blinding: Unclear<br>Data: Low<br>SR: Unclear<br>Other: Low
Rhodes[38]	Boston, United States<br>Jan 2007 –<br>Jun 2009	Singleton pregnancy, > 25 years, 13-28 weeks gestation, overweight or obese.<br>Excluded: Smoking, alcohol consumption, major illness, medications or supplements that affect body weight, high levels of physical activity, lactation in last 3 months.<br>Sample: 46 (25 low-GI, 21 low-fat)<br>Final analyses: 22 low-GI, 16 low-fat	Low-GI: Recommended energy 45% CHO, 20% protein, 35% fat.<br>Low-fat: Recommended energy 55% CHO, 20% protein, 25% fat (low saturated fat).<br>2 x 1 hour counselling sessions and maintenance visits every 2-4 weeks.<br>Duration: 12-27 weeks	Low- GI: 6.4±4.5<br>Low-fat: 6.9±4.2<br>$p = 0.74$<br>GWG:<br>Randomization - 36 weeks	Random: Unclear<br>AC: Low<br>Blinding: Unclear<br>Data: Unclear<br>SR: Unclear<br>Other: Low
Thornton[63]	New York, United States<br>Jun 1998-<br>May 2005	Singleton pregnancy, 12-28 weeks gestation, obese.<br>Excluded: diabetes, HTN, chronic renal disease.<br>Sample: 257 (133 int, 124 con)<br>Final analyses: 116 int, 116 con	Energy restricted diets to 24kcal/kg/day. Recommended energy 40% CHO, 30% protein, 30% fat.<br>Duration: 12-28 weeks	Int: 5.0±6.8<br>Con: 14.1±7.4<br>$p < 0.01$<br>GWG: Randomization - delivery	Random: Low<br>AC: Low<br>Blinding: Unclear<br>Data: Low<br>SR: Unclear<br>Other: Low
Vitolo[47]	Porto Alegre, Brazil<br>Jan 2007 –<br>May 2008	Singleton pregnancy, 10-29 weeks gestation, prenatal care group of health unit.<br>Excluded: HIV+, diabetes, HTN, anemia, >35 years old, condition requiring a special diet.<br>Sample: 315 (159 int, 162 con)<br>Final analyses: 152 int, 155 con	A counselling session based on dietary guidelines in first interview. Advice based on BMI. Women received booster advice one month after baseline.<br>Duration: 4 weeks	Healthy weight<br>Int: 461±135[e]<br>Con: 492±222[e]<br>$p = 0.2$<br>Overweight/obese<br>Int: 342±144[e]<br>Con: 420±185[e]<br>$p = 0.01$<br>GWG: Baseline – 36 weeks	Random: Low<br>AC: Unclear<br>Blinding: Unclear<br>Data: Low<br>SR: Unclear<br>Other: Unclear
Walsh[62]	Dublin, Ireland<br>Jan 2007-<br>Jan 2011	Singleton pregnancy, > 18 years, <18 weeks gestation, previously delivered an infant >4kg.<br>Excluded: Previous GDM, drug use.<br>Sample: 800 (394 int, 406 con)<br>Final analyses: 372 int, 387 con	A group education session regarding general healthy eating and low-GI foods.<br>Duration: 24 weeks	Int: 12.2±4.4<br>Con: 13.7±4.9<br>$p = 0.017$	Random: Low<br>AC: Low<br>Blinding: Unclear<br>Data: Low<br>SR: Unclear<br>Other: Low

Wolff[67]	Copenhagen, Denmark	Singleton pregnancy, > 18 years, <15 weeks gestation, obese.<br>Excluded: >45 years old, medical complications that affect foetal growth.<br>Sample: 66 (28 int, 38 con)<br>Final analyses: 23 int, 27 con	10 x 1 hour consultations with a dietitian based on Danish dietary recommendations.<br>Recommended energy 55% CHO, 15-20% protein, 30% fat.<br>Duration: 25 weeks	Int: 6.6 ±5.5<br>Con: 13.3±7.5<br>p = 0.002<br>GWG: Randomization - delivery	Random: Low<br>AC: Unclear<br>Blinding: Unclear<br>Data: Low<br>SR: Unclear<br>Other: Low

[a] Gestational weight gain (kg): mean (standard deviation) unless otherwise stated.

[b] Based on Cochrane Collaboration Risk of Bias. Random = Randomization; AC = Allocation concealment; Blinding = Blinding of participants, personal and outcome; Data = Incomplete data; SR = Selective reporting; Other = Other bias.

[c] GWG within Institute of medicine recommendations.

[d] GWG (kg) median and interquartile range

[e] Weekly rate of weight gain (grams/week)

Other acronyms and abbreviations in alphabetical order:

BMI = body mass index, Con = control group, GCT = glucose challenge test, GDM = gestational diabetes mellitus, GI = glycemic index, GWG = gestational weight gain, HTN = hypertension, Int = intervention group, OGTT = oral glucose tolerance test, SM = standard management
