# Supplementary material for: Attenuating Pregnancy Weight Gain—What Works and Why: A Systematic Review and Meta-Analysis
Source: Nutrients. 2018 Jul 22;10(7):944. doi: 10.3390/nu10070944 (PMC6073617; doi:10.3390/nu10070944)
Supplement: Supplementary file 1 [file nutrients-10-00944-s001.zip › Table S3_Summary of study characterisitics (1).pdf]

Table S3: Summary of study characteristics according to intervention type

		Diet (n = 16) n (%)	Exercise (n = 27) n (%)	Lifestyle (n = 33) n (%)	eHealth (n = 5) (n (%)	Other (n = 8)[a] n (%)	Total (n = 89) n (%)
**Commenced (Trimester)**							
	**1**	4 (25)	14 (52)	7 (21)	2 (40)	4 (50)	31 (35)
	**2**	11 (69)	13 (48)	26 (79)	3 (60)	4 (50)	57 (64)
	**3**	0 (0)	0 (0)	0 (0)	0 (0)	0 (0)	0 (0)
	**Unsure**	1 (6)	0 (0)	0 (0)	0 (0)	0 (0)	1 (1)
	**Total**	16 (100)	27 (100)	33 (100)	5 (100)	8 (100)	89 (100)
**Duration (Weeks)**							
	**<12**	0 (0)	0 (0)	2 (6)	1 (20)	0 (0)	3 (3)
	**12-29**	14 (88)	18 (67)	28 (85)	3 (60)	7 (88)	70 (79)
	**≥30**	1 (6)	9 (23)	3 (9)	1 20)	1 (13)	15 (17)
	**Unsure**	1 (6)	0 (0)	0 (0)	0 (0)	0 (0)	1 (1)
	**Total**	16 (100)	27 (100)	33 (100)	5 (100)	8 (100)	89 (100)
**Intensity (Hours)**							
	**<4**	5 (31)	2 (7)	9 (27)	1 (20)	4 (50)	21 (24)
	**4-8**	6 (34)	2 (7)	6 (18)	0 (0)	0 (0)	14 (16)
	**>8**	3 (19)	22 (81)	12 (36)	0 (0)	2 (25)	39 (44)
	**Unsure**	2 (13)	1 (4)	6 (18)	4 (80)	2 (25)	15 (17)
	**Total**	16 (100)	27 (100)	33 (100)	5 (100)	8 (100)	89 (100)
**Frequency (Contact)**							
	**≤3**	5 (31)	2 (7)	5 (15)	1 (20)	2 (25)	15 (17)
	**4-7**	4 (25)	2 (7)	9 (27)	0 (0)	1 (13)	16 (18)
	**≥8**	7 (44)	22 (81)	19 (58)	0 (0)	5 (63)	53 (60)
	**Unsure**	0 (0)	1 (4)	0 (0)	4 (80)	0 (0)	5 (6)
	**Total**	16 (100)	27 (100)	33 (100)	5 (100)	8 (100)	89 (100)
**Delivery method**							
	**Individual**	15 (94)	9 (23)	22 (67)	3 (60)	8 (100)	57 (64)
	**Group**	0 (0)	12 (44)	8 (24)	0 (0)	0 (0)	20 (2)
	**Mixed**	1 (6)	2 (7)	2 (6)	2 (40)	0 (0)	7 (8)
	**Unsure**	0 (0)	4 (15)	1 (3)	0 (0)	0 (0)	5 (6)
	**Total**	16 (100)	27 (100)	33 (100)	5 (100)	8 (100)	89 (100)

[a]Other included pharmacological, adolescent, routine weighing
