# Supplementary material for: Attenuating Pregnancy Weight Gain—What Works and Why: A Systematic Review and Meta-Analysis
Source: Nutrients. 2018 Jul 22;10(7):944. doi: 10.3390/nu10070944 (PMC6073617; doi:10.3390/nu10070944)
Supplement: Supplementary file 1 [file nutrients-10-00944-s001.zip › Table S4_ PA Interventions_Results Summary.pdf]

Table S4: Description of physical activity interventions aimed at preventing excessive gestational weight gain.

First author	Setting and date	Population	Intervention	GWG[a] and GWG measure	Quality[b]
Bacchi[86]	Buenos Aires, Argentina	Singleton and uncomplicated pregnancy.<br>Excluded: Contraindication to exercise, not planning to deliver at study hospital.<br>Sample: 140 (70 int, 70 con)<br>Final analyses: (49 int, 62 con)	Aquatic PA, 55-60 mins, 3 days per week. Aerobic and strength exercises.<br>Duration: 26 weeks	Int: 12.7±2.6<br>Con: 13.9±4.3<br>$p = 0.1$<br>GWG: First prenatal visit – 37 weeks	Random: Low<br>AC: Low<br>Blinding: Unclear<br>Data: Unclear<br>SR: Low<br>Other: Low
Barakat[72] 2013	Madrid, Spain<br>Sep 2007 – Jan 2011	Singleton pregnancy, sedentary, no contraindications to exercise.<br>Sample: 510 (255 int, 225 con)<br>Final analyses: 210 int, 218 con	Group exercise classes, 50-55 mins, 3 days per week. Aerobic, muscle strength and flexibility activities.<br>Duration: 28 weeks	Int: 11.6±3.7<br>Con: 13.3±4.1<br>$p < 0.01$<br>GWG: Pre-pregnancy - delivery	Random: Unclear<br>AC: Unclear<br>Blinding: Unclear<br>Data: Low<br>SR: High<br>Other: Unclear
Barakat[73]	Madrid, Spain	Singleton pregnancy.<br>Excluded: Not planning to give birth at study hospital, contraindications to exercise.<br>Sample: 80 (40 int, 40 con)<br>Final analyses: 34 int, 33 con	Group exercise classes, 35-45 mins, 3 days per week. Warm-up, core session of toning and very light resistance exercise, and cool-down.<br>Duration: 33 weeks	Int: 11.9±3.1<br>Con: 13.9±2.1<br>$p < 0.03$<br>GWG: Pre-pregnancy - delivery	Random: Low<br>AC: Unclear<br>Blinding: Unclear<br>Data: Low<br>SR: Unclear<br>Other: Unclear
Barakat[70]	Madrid, Spain<br>Jan 2000 - Mar 2002	Singleton pregnancy, sedentary, no risk of preterm delivery, no contraindications to exercise.<br>Sample: 160 (80 int, 80 con)<br>Final analyses: 72 int, 70 con	Group exercise classes, 35-40 mins, 3 days per week. Sessions were individually controlled and consisted of light-moderate PA.<br>Duration: 26 weeks	Int: 11.5±3.7<br>Con: 12.4±3.4<br>$p > 0.1$<br>GWG: Baseline - delivery	Random: Unclear<br>AC: Unclear<br>Blinding: Unclear<br>Data: Unclear<br>SR: High<br>Other: Unclear
Barakat[71]	Madrid, Spain<br>Dec 2011 – Jan 2015	Singleton pregnancy.<br>Excluded: diabetes or GDM at baseline, risk of preterm delivery.<br>Sample: 840 (420 int, 420 con)<br>Final analyses: 382 int 383 con	Group exercise classes, 50-55 mins, 3 days per week. Warm-up, core session of moderate resistance exercise, and cool-down.<br>Duration: 30 weeks	Int: 12.1±3.7<br>Con: 12.9±4.5<br>$p = 0.01$<br>GWG: Pre-pregnancy – last weight before delivery	Random: Low<br>AC: Unclear<br>Blinding: Unclear<br>Data: Low<br>SR: Unclear<br>Other: Unclear
Barakat[69]	Madrid, Spain	Singleton pregnancy<br>Excluded: Contraindications such as CVD, risk of premature labour, diabetes.<br>Sample: 100 (50 int, 50 con)<br>Final analyses: 40 int, 43 con	Group exercise classes, 35-45 mins, 3 days per week, 2 x land-based and 1 aquatic. Warm- up, core session of light resistance activities, and cool-down.<br>Duration: 30 weeks	Int: 12.5±3.2<br>Con: 13.8±3.1<br>$p > 0.05$<br>GWG: Pre-pregnancy - delivery	Random: Unclear<br>AC: Unclear<br>Blinding: Unclear<br>Data: Low<br>SR: Unclear<br>Other: Unclear

Barakat[74]	Madrid,<br>Spain	Singleton pregnancy.<br>Excluded: Contraindications to exercise.<br>Sample: 320 (160 int, 160 con)<br>Final analyses: 138 int 152 con	Group exercise classes, 55-60 mins, 3 days per week. Warm- up, core session of toning<br>and resistance activities, and cool-down.<br>Duration: 30 weeks	Int: 11.9±3.7<br>Con: 13.7±4.1<br>$p < 0.01$<br>GWG: Pre-pregnancy - delivery	Random: Low<br>AC : Unclear<br>Blinding: Unclear<br>Data: Low<br>SR: High<br>Other: Unclear
Barakat[87]	Madrid<br>Spain<br>Mar 2014 – Jan 2017	Singleton and uncomplicated pregnancy.<br>Excluded: Contraindications to exercise, not planning to deliver at study hospital.<br>Sample: 508 (255 int, 253 con)<br>Final analysis: 176 int, 149 con	Supervised PA program, 55-60 mins, 3 days per week consisting of aerobic and resistance exercises.<br>Duration: 30 weeks	Int: 12.0±3.3<br>Con: 12.8±3.9<br>$p = 0.1$<br>GWG: Baseline – 37 weeks	Random: Low<br>AC: Low<br>Blinding: Unclear<br>Data: Unclear<br>SR: Low<br>Other: Unclear
Bisson[82]	Quebec, Canada<br>Oct 2011 -<br>Nov 2013	Singleton pregnancy, > 18 years, obese.<br>Excluded: Contraindications to exercise, a high level of pre-gestational exercise.<br>Sample: 50 (25 int, 25 con)<br>Final analyses: 23 int, 22 con	A 12-week, supervised exercise program at a hospital-based centre. Participants were individually supervised once a week and invited to 2 more sessions per week. On non-training days women were encouraged to be as active as possible.<br>Duration: 12 weeks	Int: 12.3±4.0<br>Con: 12.2±5.9<br>$p = 0.03$<br>GWG: Pre-pregnancy - delivery	Random: Low<br>AC: Low<br>Blinding: Unclear<br>Data: Low<br>SR: Low<br>Other: Low
Cavalcante[78]	Sao Paulo,<br>Brazil<br>Mar 2002 -<br>Nov 2004	Singleton pregnancy, 16-20 weeks gestation, sedentary.<br>Excluded: > 2 caesarean births, contraindication to exercise.<br>Sample: 71 (34 int 37 con)<br>Final analyses: 34 int, 37 con	Water aerobic classes, 50 mins, 3 days per week. Sessions were in an indoor swimming pool and involved moderate-intensity exercises so that women worked at 70% capacity.<br>Duration: 20-24 weeks	Int: 14.3±2.1<br>Con: 15.1±1.6<br>$p = 0.38$<br>GWG: Pre-pregnancy - delivery	Random: Low<br>AC: Low<br>Blinding: Unclear<br>Data: Unclear<br>SR: Unclear<br>Other: High
Clapp[76]	Ohio, United States	Singleton pregnancy, enrolled before conception.<br>Excluded: Substance use, premature labour, HTN, GDM, IUGR, failure to maintain exercise regimen.<br>Sample: 46 randomized<br>Final analyses: 22 int, 24 con	Weight-bearing exercise, 20 minutes, three to five times per week at an intensity between 55-60% of capacity.<br>Duration: 30 weeks	Int: 15.7±4.7<br>Con: 16.3±3.4<br>*Not significant*<br>GWG: Pre-pregnancy - delivery	Random: Unclear<br>AC: Unclear<br>Blinding: Unclear<br>Data: Low<br>SR: Unclear<br>Other: Unclear

Clapp[34]	Ohio,<br>United<br>States	Singleton pregnancy, enrolled before conception, regularly exercising.<br>Excluded: Substance use, premature labour, mid-trimester bleeding, HTN, GDM, IUGR, failure to maintain exercise regimen.<br>Sample: 80 randomized<br>Final analyses: 26 Lo-Hi, 24 Mod-Mod, 25 Hi-Lo	*Lo-Hi:* Weight-bearing exercise (treadmill, step aerobics or stair stepper) 20 mins, 5 days per week until week 20, then gradually increasing to 60 mins by week 24 and maintain until the end of pregnancy.<br>*Mod-Mod:* Weight-bearing exercise (treadmill, step aerobics or stair stepper) 40 mins, 5 days per week from 8 weeks to end of pregnancy<br>*Hi-Lo:* Weight-bearing exercise (treadmill, step aerobics or stair stepper) 60 mins, 5 days per week until week 20, then gradually decreasing to 20 mins by the end of pregnancy.<br>Duration: 32 weeks	Lo-Hi: 12.0±4.1<br>Mod-Mod: 14.6±4.4<br>Hi-Lo: 15.5±4.5<br>*GWG was significantly less in the*<br>*Lo-Hi group*<br>$p < 0.02$<br>GWG: Pre-pregnancy - delivery	Random: Unclear<br>AC: Unclear<br>Blinding: Unclear<br>Data: Unclear<br>SR: Unclear<br>Other: Unclear
Daly[89]	Dublin,<br>Ireland<br>Nov 2013 – Apr 2016	Singleton pregnancy, > 18 years, <17 weeks gestation, BMI > 30, English speaking.<br>Excluded: Pre-existing diabetes, HTN, medications affecting insulin sensitivity<br>Sample: 88 randomized<br>Final analyses: 34 int, 42 con	Supervised exercise classes, 50-60 mins, 3 days per week.<br>Duration: 27 weeks	Int: 6.2±6.0<br>Con: 7.9±4.8<br>$p = 0.15$<br>GWG: Baseline - 36 weeks	Random: Low<br>AC: Low<br>Blinding: Unclear<br>Data: Unclear<br>SR: High<br>Other: Low
Da Silva[88]	Pelostas,<br>Brazil<br>Apr 2014 – Mar 2016	Singleton pregnancy, > 18 years, physically inactive.<br>Excluded: HTN, CVD, diabetes, history of miscarriage, preterm birth, BMI > 35, heavy smoker.<br>Sample: 639 (213 int, 426 con)<br>Final analyses: 85 int, 407 con	Structured, individually supervised, moderate intensity exercise program, 60 mins, 3 days per week.<br>Duration: 24 weeks	Int: 12.7±5.7<br>Con: 12.9±6.5<br>$p = 0.2$<br>GWG: Baseline – 36 weeks	Random: Low<br>AC: Unclear<br>Blinding: Unclear<br>Data: Unclear<br>SR: Low<br>Other: Unclear
Dekker Nitert[84]	Brisbane,<br>Australia	Singleton pregnancy, >12 weeks gestation, obese.<br>Excluded: Contraindication to exercise, non-English speaking, type 1 diabetes, anemia, heart or lung disease, cerclage, hyperthyroidism, poorly controlled seizures, heavy smoker.<br>Sample: 50 randomized<br>Final analyses: 19 int, 16 con	At baseline, all participants attended a group education session regarding exercise, nutrition and GWG. Women were given an individualised exercise plan and attended monthly face-to-face consultations with physiotherapists.<br>Duration: 26 weeks	Int: 7.9±4.0<br>Con: 8.3±6.1<br>$p = 0.81$<br>GWG: Baseline – 36 weeks	Random: Unclear<br>AC: Unclear<br>Blinding: Unclear<br>Data: High<br>SR: Low<br>Other: Unclear
Garnaes[90]	Trondheim,<br>Norway<br>Sept 2010 – Mar 2015	Singleton pregnancy, >18 years, <18 weeks gestation, BMI > 28.<br>Excluded: Preterm labour risk, disease that could interfere with the intervention, habitual exercise.	Supervised exercise classes, 60 mins, 3 days per week. In addition, women asked to follow a home-based 50-min program 1 day per week.<br>Duration: 23 weeks	Int: 10.5±0.8<br>Con: 9.2±1.2<br>$p = 0.35$<br>GWG: Baseline - delivery	Random: Low<br>AC: Low<br>Blinding: Unclear<br>Data: Low<br>SR: Unclear

		Sample: 91 (46 int, 45 con)<br>Final analyses: 38 int, 36 con			Other: Low
Garshasbi[77]	Tehran, Iran<br>Apr 2003 -<br>Jan 2004	Singleton pregnancy, 20-28 years, 17-22 weeks gestation, housewives or high-school graduates.<br>Excluded: Contraindications to aerobic exercise, exercise before pregnancy, orthopaedic disease.<br>Sample: 266 (161 int, 105 con)<br>Final analyses: 107 int, 105 con	60 min program for strengthening abdominal muscles consisting of 15 movements, 3 days per week. Supervised by a midwife.<br>Duration: 18-23 weeks	Int: 14.1±3.8<br>Con: 13.8±5.2<br>$p = 0.63$	Random: Unclear<br>AC: Low<br>Blinding: Unclear<br>Data: High<br>SR: Unclear<br>Other: Unclear
Haakstad[79]	Oslo, Norway<br>Sep 2007 -<br>Mar 2008	Singleton pregnancy, < 24 weeks gestation, nulliparous, not in exercise program in the 6 months before pregnancy.<br>Excluded: > 2 miscarriages, heart disease, persistent bleeding after 12 weeks, poorly controlled thyroid disease, HTN, pre-eclampsia.<br>Sample: 105 (52 int, 53 con)<br>Final analyses: 14 int, 53 con	A 12-week program of 60 mins supervised dance classes, at least twice a week. Women advised to participate in moderate PA of on remaining days.<br>Duration: 12 weeks	Int: 11.0±2.0<br>Con: 13.8± 4.0<br>$p = 0.01$<br>GWG: Pre-pregnancy– 36 weeks	Random: Low<br>AC: Unclear<br>Blinding: Low<br>Data: Low<br>SR: Unclear<br>Other: Low
Kong[81]	Iowa, United States	Singleton pregnancy, 18-45 years, overweight or obese, < 3, 30 min sessions of PA 6 months before enrolment.<br>Excluded: History of chronic disease such as CVD, thyroid disease, prior GDM, smoking.<br>Sample: 42 (19 int, 23 con)<br>Final analyses: 18 int, 19 con	An unsupervised walking program consisting of a safety training session regarding 30 mins of leisure activity according to guidelines. Women were given a treadmill for intervention and encouraged to walk in any setting.<br>Duration: 22 weeks	Overweight int: 10.5±5.4<br>Overweight con: 9.9±6.1<br>*Not significant*<br>Obese int: 12.1±9.0<br>Obese con: 12.5±8.5<br>*Not significant*<br>GWG: Baseline – 36 weeks	Random: Low<br>AC: Low<br>Blinding: Low<br>Data: Low<br>SR: Unclear<br>Other: Low
Nascimento[80]	Sao Paulo, Brazil<br>Aug 2008-<br>Mar 2010	Singleton pregnancy, ≥18 years, 14-24 weeks gestation, BMI ≥26kg/m^2.<br>Excluded: multiple gestations, contraindications to exercise.<br>Sample: 82 (40 int, 42 con)<br>Final analyses: 39 int, 41 con	A weekly group exercise class consisting of stretching, upper and lower limb strengthening and relaxation. Women were encouraged to walk 5 days per week.<br>Duration: 20 weeks	Int: 10.3±5.0<br>Con: 11.5±7.4<br>$p = 0.54$<br>GWG: Pre-pregnancy - 38/39 weeks	Random: Low<br>AC: Unclear<br>Blinding: Unclear<br>Data: Low<br>SR: Unclear<br>Other: Unclear
Nobles[35]	Massachusetts, United States<br>2007 – 2012	High risk of GDM<br>Excluded: Already physically active, <18 years and >40 years, >20 weeks, unable to read English.<br>Sample: 290 (143 PA, 147 H&W)<br>Final analyses: 118 PA, 123 H&W	PA: Face-to-face counselling at baseline, followed by weekly and bi-weekly phone calls with tailored PA advice<br>H&W: Mailings of brochures related to general pregnancy health and wellbeing.<br>Duration: 12 weeks	PA: 12.9±8.3<br>H&W: 13.8±9.0<br>$p = 0.39$<br>GWG: Pre-pregnancy - delivery	Random: Unclear<br>AC: Unclear<br>Blinding: Unclear<br>Data: Low<br>SR: Unclear<br>Other: Unclear

Oostdam[85]	Amsterdam<br>Netherlands<br>Jan 2007 -<br>Jan 2011	Singleton pregnancy, overweight or obese with previous GDM, macrosomia or first-grade relative with T2DM.<br>Excluded: > 20 weeks gestation, < 18 years, GDM, HTN, alcohol or drug use, other health complications.<br>Sample: 121 (62 int, 59 con)<br>Final analyses (ITT): 40 int, 45 con	2 x 60 minute PA sessions with aerobic and strength exercises supervised by a physiotherapist at participating hospital.<br>Duration: 25 weeks (women also followed up 12 weeks post-partum).	Int: 6.2±5.0[c]<br>Con: 5.6±3.5[c]<br>*Not significant*<br>GWG: Baseline - 32 weeks	Random: Low<br>AC: Low<br>Blinding: Unclear<br>Data: Unclear<br>SR: Low<br>Other: Low
Perales[45]	Madrid, Spain<br>Oct 2009 -<br>Jan 2013	Singleton pregnancy, overweight or obese.<br>Excluded: Obstetric complications.<br>Sample: 129 (65 int, 64 con)<br>Final analyses: 52 int, 54 con	Group exercise program, 55-60 mins, three times per week. Warm up, core aerobic activities, and cool down.<br>Duration: 30 weeks	Int: 46.8%[d]<br>Con: 67.3%[d]<br>$p = 0.03$<br>GWG: Pre-pregnancy - delivery.	Random: Low<br>AC: Unclear<br>Blinding: Unclear<br>Data: Low<br>SR: low<br>Other: Unclear
Rodriguez-Blanque[92]	Granada, Spain<br>Mar 2016 – Apr 2016	Singleton pregnancy, 12-20 weeks.<br>Excluded: Contraindications to PA<br>Sample: 140 (70 int, 70 con)<br>Final analyses: 65 int, 65 con	Aquatic PA sessions, 60 mins, 3 days/week.<br>Duration: 17 weeks	Int: 8.3±2.8<br>Con: 11.2±3.5<br>$p < 0.01$<br>GWG: 12 – 36 weeks	Random: Low<br>AC: Unclear<br>Blinding: Unclear<br>Data: Unclear<br>SR: Unclear<br>Other: Unclear
Ronnberg[83]	Örebro, Sweden<br>Jun 2007 -<br>Mar 2010	Singleton pregnancy, ≥18 years old, <16 weeks gestation.<br>Excluded: BMI <19kg/m^2, history of an eating disorder, obstetric complications, IUGR, chronic illness.<br>Sample: 445 (221 int 224 con)<br>Final analyses: 192 int, 182 con	Individual education session regarding IOM GWG guidelines and PA prescription issued by a midwife. Weight gain monitored on chart.<br>Duration: 30 weeks	Int: 14.2±4.5<br>Con: 15.3±5.4<br>$p = 0.029$<br>GWG: First pre-natal visit - delivery	Random: Unclear<br>AC: Unclear<br>Blinding: Unclear<br>Data: High<br>SR: Unclear<br>Other: Unclear
Ruiz[75]	Madrid, Spain<br>Sep 2007 -<br>Jan 2011	Singleton pregnancy, sedentary, not at risk of preterm delivery.<br>Excluded: Contraindication to exercise<br>Sample: 962 (481 int, 481 con)<br>Final analyses: 481 int, 481 con	Group exercise classes, 55-60 mins, 3 days per week. Session consisted of a warm-up, core session of moderate intensity aerobic and resistance exercises, and cool-down.<br>Duration: 30 weeks	Int: 11.9±3.8<br>Con: 13.2±4.3<br>$p < 0.01$<br>GWG: First prenatal visit - delivery	Random: Low<br>AC: Unclear<br>Blinding: Unclear<br>Data: Low<br>SR: Low<br>Other: Low
Wang[91]	Peking, China<br>Dec 2014 –<br>Jul 2016	Singleton pregnancy, >18 years, <12 weeks gestation, BMI > 24, non-smoker.<br>Excluded: Cervical insufficiency, pre-existing chronic disease, metformin, corticosteroids.<br>Sample: 300 (150 int, 150 con)<br>Final analyses: 112 int, 114 con	Supervised cycling program, 30 mins, 3 days per week.<br>Duration: 27 weeks	Int: 8.4±3.7<br>Con: 10.5±3.3<br>$p < 0.01$<br>GWG: Baseline - delivery	Random: Low<br>AC: Low<br>Blinding: Unclear<br>Data: Low<br>SR: Unclear<br>Other: Unclear

[a] Gestational weight gain (kg): mean (standard deviation) unless otherwise stated.

[b]Based on Cochrane Collaboration Risk of Bias. Random = Randomization; AC = Allocation concealment; Blinding = Blinding of participants, personal and outcome; Data = Incomplete data; SR = Selective reporting; Other = Other bias.

[c] Intention to treat data.

[d] Percentage that experienced excessive GWG according to Institute of Medicine recommendations.

Other acronyms and abbreviations in alphabetical order: BMI = body mass index, Con = control group, CVD = cardiovascular disease, GDM = gestational diabetes mellitus, GWG = gestational weight gain, Hi = high, HTN = hypertension, Int = intervention group, IOM = Institute of Medicine, IUGR = intrauterine growth restriction, Lo = low, PA = Physical Activity, T2DM = type 2 diabetes mellitus.
