# Supplementary material for: Attenuating Pregnancy Weight Gain—What Works and Why: A Systematic Review and Meta-Analysis
Source: Nutrients. 2018 Jul 22;10(7):944. doi: 10.3390/nu10070944 (PMC6073617; doi:10.3390/nu10070944)
Supplement: Supplementary file 1 [file nutrients-10-00944-s001.zip › Table S5_ LS Interventions_Results Summary.pdf]

Table S5: Description of lifestyle interventions aimed at preventing excessive gestational weight gain.

First author	Setting and date	Population	Intervention	GWG[a] and GWG measure	Quality[b]
Althuizen[111]	Netherlands<br>Feb 2005 –<br>May 2006	Singleton pregnancy, < 14 weeks gestation, nulliparous, literate in Dutch.<br>Sample: 246 (123 int, 123 con)<br>Final analyses (GWG only):106 int, 113 con	Four face-to-face counselling sessions regarding weight, PA and diet (8, 22, 30 and 36 weeks).<br>Duration: 30 weeks (including 8 weeks post-partum)	Int: 11.6±4.1<br>Con: 11.1±3.2<br>*Not significant*<br>GWG: 15 – 35 weeks	Random: Low<br>AC: Unclear<br>Blinding: Unclear<br>Data: Low<br>SR: High<br>Other: Unclear
Asbee[97]	Charlotte, United States<br>Oct 2005 –<br>Apr 2007	Singleton pregnancy, 18-49 years, prenatal care established at 6-16 weeks gestation, English or Spanish speaking.<br>Excluded: BMI > 40, pre-existing medical conditions.<br>Sample: 144 randomized<br>Final analyses: 57 int, 43 con	Counselling session regarding diet, PA and GWG at baseline. Recommended energy 40% CHO, 30% protein, 30% fat, and 3-5 moderate PA sessions per week. Women weighed at routine appointments and given further advice if not meeting guidelines.<br>Duration: 26 weeks	Int: 12.6±5.7<br>Con: 16.2±7.0<br>$p = 0.01$<br>GWG: Pre- pregnancy - delivery	Random: Low<br>AC: Low<br>Blinding: Unclear<br>Data: High<br>SR: Unclear<br>Other: Unclear
Asci[98]	Istanbul, Turkey<br>Jun 2011 –<br>Jul 2012	Singleton pregnancy, > 18 years old, < 12 weeks gestation, no intention to lose weight during pregnancy.<br>Sample: 102 (51 int, 51 con)<br>Final analyses: 45 int, 45 con	One hour interviews regarding: GWG (weeks 12-15), PA (weeks 16-18), nutrition (weeks 20-24) and weight (week 37). Women given GWG card at baseline and weight was taken at each meeting.<br>Duration: 25 weeks	Int: 12.5±5.0<br>Con: 12.3±4.8<br>$p = 0.87$<br>GWG: Pre-pregnancy – 37 weeks	Random: Low<br>AC: Unclear<br>Blinding: Unclear<br>Data: Unclear<br>SR: Unclear<br>Other: Unclear
Bogaerts[104]	Flanders, Belgium<br>Mar 2008 –<br>Apr 2011	Singleton pregnancy, < 15 weeks gestation, obese, literate in Dutch.<br>Excluded: Type 1 diabetes, primary need for nutrition advice.<br>Sample: 205 (64 brochure, 78 LS, 63 con)<br>Final analyses: 58 brochure, 76 LS, 63 con	Brochure: A purpose-designed brochure regarding diet, PA and limiting excessive GWG.<br>LS: Four 1 ½ - 2 hour group sessions regarding energy intake. Recommended energy 50-55% CHO, 9-11% protein, 30-35% fat. Motivational interviewing, food diaries and label reading used (in addition to brochure).<br>Duration: 25 weeks	Brochure: 9.5±6.8<br>LS: 10.6±7.0<br>Con: 13.5±7.3<br>$p < 0.01$<br>GWG: Pre-pregnancy - delivery	Random: Low<br>AC: Low<br>Blinding: Unclear<br>Data: Low<br>SR: Unclear<br>Other: Low
Bruno[116]	Modena, Italy<br>Feb 2013 –<br>Jun 2014	Singleton pregnancy, > 18 years, BMI > 25.<br>Excluded: Chronic disease, medications that affect weight, bariatric surgery, previous GDM, contraindications to exercise.<br>Sample: 191 (96 int, 95 con)<br>Final analyses: 69 int, 62 con	One hour counselling session with dietitian at enrolment with tailored dietary advice. PA advice was to participate in 30 mins PA 3 days per week.<br>Duration: 30 weeks	Int:9.5±6.4<br>Con: 9.1±6.7<br>$p = 0.75$<br>GWG: Baseline – 36 weeks	Random: Low<br>AC: Low<br>Blinding: Unclear<br>Data: Unclear<br>SR: Low<br>Other: Low
Cahill[39]	Missouri, United States<br>Oct 2012 –<br>Mar 2016	African American ancestry, singleton pregnancy, 18-45 years, <15 weeks, BMI 25-45, disadvantaged socioeconomic status	PAT: One-on-one home visits with parent educators every 2 weeks. Focus on development-centred parenting support and education.	PAT: 9.9±5.3<br>PAT+: 8.1±5.6<br>$p = 0.01$<br>GWG: 15 – 35 weeks	Random: Low<br>AC: Unclear<br>Blinding: Unclear<br>Data: Low

		Excluded: Diabetes, history of GDM or macrosomia, contraindications to exercise, substance abuse, non-English speaking.<br>Sample: 267 (134 PAT, 133 PAT+)<br>Final analyses: 121 PAT, 119 PATE+	PAT+: PAT curriculum plus lifestyle curriculum based on cognitive behaviour therapy to achieve optimal GWG.<br>Duration: 20 weeks		SR: Unclear<br>Other Unclear
Dodd[112]	Adelaide, Australia<br>Jun 2008 – Dec 2011	Singleton pregnancy, 10 – 20 weeks gestation, overweight or obese.<br>Excluded: Pre-existing diabetes<br>Sample: 2212 (1108 int, 1104 con)<br>Final analyses: 1080 int, 1072 con	Three face-to face meetings and three phone calls consisting of dietary and LS advice. Women were provided with meal plans, recipes and snack ideas.<br>Duration: 22 weeks	Overall:<br>Int: 9.4±5.7<br>Con: 9.4±5.8<br>*p* = 0.89	Random: Low<br>AC: Low<br>Blinding: Low<br>Data: Unclear<br>SR: Low<br>Other: Low
Gesell[108]	Nashville, United States<br>Jan 2011 – Apr 2011	Singleton pregnancy, > 16 years old, 10 – 28 weeks gestation, literate in English or Spanish<br>Sample: 135 (68 int, 67 con)<br>Final analyses: 38 int, 36 con	12 weekly, 90 min group sessions based on social learning theory. Topics included nutrition PA, sleep, weight monitoring, goal setting, cooking skills, money management and anxiety.<br>Duration: 12 weeks	Int: 8.9±5.6<br>Con: 10.2±7.1<br>*p* = 0.37<br>GWG: Pre-pregnancy - delivery	Random: Low<br>AC: Low<br>Blinding: Unclear<br>Data: Low<br>SR: Unclear<br>Other: Low
Guelinckx[99]	Leuven, Belgium<br>Mar 2006 – Jan 2008	Singleton pregnancy, > 15 weeks gestation, obese, literate in Dutch.<br>Excluded: Pre-existing diabetes, GDM, metabolic disorders, Chrons disease, allergies, preterm birth.<br>Sample: 195 (65 passive, 65 active, 65 con)<br>Final analyses: 37 passive, 42 active, 43 con	Passive: A brochure regarding diet, PA and GWG.<br>Active: Three 1-hour group sessions. Recommended energy 55% CHO, 9-11% protein, 30-35% fat.<br>Participants advised on label reading, PA, emotional eating.<br>Duration: 25 weeks	Passive: 10.9 ±5.6<br>Active: 9.8±7.6<br>Con: 10.6±6.9<br>*Not significant*<br>GWG: Pre-pregnancy - delivery	Random: Unclear<br>AC: Unclear<br>Blinding: Unclear<br>Data: Low<br>SR: Unclear<br>Other: Low
Harrison[105]	Melbourne, Australia	Singleton pregnancy, 12 – 15 weeks gestation, overweight or obese, high risk of GDM.<br>Excluded: Diabetes, pre-existing medical conditions, BMI > 45.<br>Sample: 228 (121 int, 107 con)<br>Final analyses: 106 int, 97 con	Four individual behaviour change sessions based on social cognitive theory. Recommendations made for diet, PA, goal setting and self-monitoring.<br>Duration: 14 weeks	Int: 6.0±2.8<br>Con: 6.9±3.3<br>*p* = 0.05<br>GWG: Randomization - 28 weeks	Random: Low<br>AC: Low<br>Blinding: Unclear<br>Data: Low<br>SR: Unclear<br>Other: Low
Hawkins[106]	Massachusetts, United States<br>Apr 2010 – Aug 2011	Singleton pregnancy, 18 – 40 years old, < 18 weeks gestation, overweight or obese, < 30 minutes PA per week.<br>Excluded: Type 2 diabetes, heart or renal disease, contraindications to moderate PA.<br>Sample: 68 (33 int, 35 con)<br>Final analyses: 32 int, 34 con	6 face-to-face counselling sessions with 5 phone calls regarding diet and PA.<br>Duration: 20-26 weeks	Int: 17.7±1.0<br>Con: 17.9±0.1<br>*p* = 0.89<br>GWG: Pre-pregnancy - delivery	Random: Unclear<br>AC: Unclear<br>Blinding: Unclear<br>Data: Low<br>SR: Unclear<br>Other: Low

Huang[100]	Taiwan<br>Jan 2006 –<br>Jun 2006	Singleton pregnancy, >18 years old, <16 weeks gestation, literate in Chinese.<br>Excluded: Cognitive impairment, psychiatric illness.<br>Sample: 240 (80 int 1, 80 int 2, 80 con)<br>Final analyses: 61 int 1, 64 int 2, 64 con	Int 1: 6 x one-to-one counselling sessions regarding diet and PA.<br>Int 2: Educational session 24-28 hours post-partum and 2 x one-to-one booster sessions at 6 and 12 weeks post-partum.<br>Duration: 24 weeks of pregnancy (post-partum not included).	Int 1: 14.0±2.4<br>Con: 16.2±3.3<br>$p < 0.01$	Random: Low<br>AC: Unclear<br>Blinding: Unclear<br>Data: Unclear<br>SR: Unclear<br>Other: Low
Hui[101]	Manitoba, Canada<br>Jul 2004 –<br>Feb 2010	Singleton pregnancy, < 26 weeks gestation.<br>Excluded: diabetes, contraindications to PA.<br>Sample: 224 (112 int, 112 con)<br>Final analyses: 102 int, 88 con	LS advice regarding PA and diet. Group PA sessions at local gym 3-5 times per week. 2 x dietary and<br>GWG counselling sessions provided at enrolment and 2 months after enrolment.<br>Duration: 10-16 weeks	Int: 14.1±6.0<br>Con: 15.2±5.9<br>$p = 0.28$<br>GWG: Pre-pregnancy - delivery<br>delivery	Random: Low<br>AC: Low<br>Blinding: Unclear<br>Data: Low<br>SR: Unclear<br>Other: Low
Hui[102]	Manitoba, Canada<br>May 2009 –<br>Dec 2011	Singleton pregnancy, < 20 weeks gestation.<br>Excluded: diabetes, contraindications to PA<br>Sample: 113 (57 int, 56 con)<br>Final analyses: 57 int, 56 con	LS advice regarding PA and diet. Group PA sessions 3-5 times per week. 2 x dietary and GWG counselling sessions provided at enrolment and 2 months after enrolment.<br>Duration: 27 weeks	Healthy weight int: 12.9±3.7<br>Healthy weight con: 16.2±4.4<br>$p = 0.03$<br>Overweight/obese int: 15.2±7.5<br>Overweight/obese con: 14.4±7.1<br>$p = 0.26$<br>GWG: Pre-pregnancy – delivery	Random: Low<br>AC: Low<br>Blinding: Unclear<br>Data: Low<br>SR: Unclear<br>Other: Low
Jing[114]	Chengdu, China<br>Sep 2012 –<br>Sep 2013	Singleton pregnancy, > 18 years.<br>Excluded: diabetes, pregnancy complications.<br>Sample: 262 (131 int, 131 con)<br>Final analyses: 115 int, 106 con	One 20 min health education session regarding GWG and GDM. Manuals were given regarding diet PA. Participants could contact a trained graduate to ask questions and receive feedback at 16-20 weeks and 20-24 weeks.<br>Duration: 12 weeks	Int: 9.2±4.0<br>Con: 9.7±3.9<br>$p = 0.40$<br>GWG: Baseline – 24 weeks	Random: Low<br>AC: Unclear<br>Blinding: Unclear<br>Data: Low<br>SR: Unclear<br>Other: Unclear
Luoto[115]	Piranmaa, Finland<br>Oct 2007 –<br>Dec 2008	Singleton pregnancy, at least one risk factor for GDM.<br>Excluded: Abnormal baseline OGTT, pre-pregnancy diabetes, contraindications to PA, substance abuse, psychiatric illness.<br>Sample: 446 (246 int, 196 con)<br>Final analyses: 216 int, 179 con	LS advice regarding PA and diet in regular antenatal visits. PA counselling focused on increasing leisure time. Diet counselling based on the Finnish dietary recommendations.<br>Duration: 29 weeks	Int: 13.8±5.8<br>Con: 14.2±5.1<br>$p = 0.52$<br>GWG: Pre-pregnancy - delivery	Random: Low<br>AC: Unclear<br>Blinding: Unclear<br>Data: Unclear<br>SR: Low<br>Other: Low
Pawalia[40]	Hisar, India<br>Jun 2016 –<br>Sep 2017	Singleton pregnancy, >18 years, <16 weeks, BMI > 18.5, mobile phone.<br>Excluded: Declared unfit by obstetrician.<br>Sample: 36 (12 PA, 12 PA+Diet, 12 con)<br>Final analyses: NR	PA: 60-min hospital-based exercise sessions, 2 days per week. Women encouraged to walk 4 days/week.<br>PA+Diet: PA intervention plus regular text messages about nutrition.<br>Duration: 27 weeks	PA: 12.4±3.9<br>PA+Diet: 12.9±3.7<br>Con:13.3±5.3<br>*Significance not reported*<br>GWG: Pre-pregnancy – delivery	Random: High<br>AC: Unclear<br>Blinding: Unclear<br>Data: High<br>SR: High<br>Other: High

Petrella[93]	Modena, Italy<br>Apr 2011 –<br>Oct 2011	Singleton pregnancy, > 18 years old, overweight or obese.<br>Excluded: chronic diseases, previous GDM, smokers, bariatric surgery, regular PA, supplements known to affect body weight.<br>Sample: 63 (33 int, 30 con)<br>Final analyses: 33 int, 28 con	A one-hour counselling session regarding an energy restricted diet: 1500kcal/day + 200 kcal/day for obese or 300kcal/day overweight. Women were also encouraged to exercise 30 minutes, 3 days per week.<br>Duration: 27 weeks	Int: 8.8±6.5<br>Con: 10.4±5.0<br>*Not significant*<br>GWG: Pre-pregnancy - delivery	Random: Low<br>AC: Low<br>Blinding: Unclear<br>Data: Unclear<br>SR: Unclear<br>Other: Low
Phelan[94]	Rhode Island, United States<br>2006 - 2008	Singleton pregnancy, > 18 years old, 10-16 weeks gestation, BMI 19.8 - 40kg/m^2, non-smoker.<br>Excluded: major health or psychiatric disease, weight-loss during pregnancy, history of ≥3 miscarriages.<br>Sample: 401 (201 int, 200 con)<br>Final analyses: 174 int, 169 con	One session regarding GWG, PA and energy intake (20kcal/kg/day).Emphasis placed on self-monitoring. Food diaries, scales and pedometer provided and women received supportive phone calls throughout pregnancy.<br>Duration: 27 weeks	Healthy weight int: 15.3±4.4<br>Healthy weight con: 16.2±4.6<br>*Significance not reported*<br>Overweight/obese int: 14.7±6.9<br>Overweight/obese con: 15.1±7.5<br>*Significance not reported*<br>GWG: Pre-pregnancy – delivery	Random: Low<br>AC: Low<br>Blinding: Unclear<br>Data: Low<br>SR: Unclear<br>Other: Low
Phelan[41]	United States<br>Nov 2012 – Oct 2015	Singleton pregnancy, 6-16 weeks, > 18 years, BMI > 25, English or Spanish speaking<br>Excluded: $HbA1_c$ > 6.5, self-reported health issues, contraindication to exercise.<br>Sample: 264 (132 int, 132 EUC)<br>Final analyses: 129 int, 127 EUC.	Int: Face-to-face counselling, 20 mins, every two weeks or monthly. If not meeting GWG target, further counselling received.<br>EUC: Enhanced usual care received a 20 min welcome visit and newsletters about health-related topics every 2 months.<br>Duration: 27 weeks	Int: 9.4±6.9<br>EUC:11.2±7.0<br>$p = 0.03$<br>GWG: Pre-pregnancy – delivery	Random: Low<br>AC: Unclear<br>Blinding: Unclear<br>Data: Low<br>SR: Low<br>Other: Unclear
Polley[95]	Pittsburgh, United States	Singleton pregnancy, >18 years of age, < 20 weeks gestation.<br>Excluded: underweight, high risk pregnancy.<br>Sample: 120 (61 int, 59 con)<br>Final analyses: 57 int, 53 con	Lifestyle advice offered at regular clinic visits regarding GWG, PA and diet. Between each visit women were mailed newsletters and contacted by phone. Women with excessive GWG were provided with extra counselling.<br>Duration: 24 weeks	Healthy weight int: 15.4±7.1<br>Healthy weight con: 16.4±4.8<br>*Not significant*<br>Overweight/obese int: 13.6±7.2<br>Overweight/obese con: 10.1±6.2<br>$p < 0.01$<br>GWG: Pre-pregnancy - delivery	Random: Unclear<br>AC: Unclear<br>Blinding: Unclear<br>Data: Low<br>SR: Unclear<br>Other: Low
Poston[96]	United Kingdom	Singleton pregnancy, > 16 years, 15 – 18 weeks gestation, obese.<br>Excluded: Underlying disorders or prescribed metformin.<br>Sample: 1555 (783 int, 772 con)<br>Final analysis: 629 int, 651 con	Women received advice on self-monitoring, problem solving, barriers to behaviour change, and enlisting social support. Women set goals regarding PA and diet, received a PA DVD and were encouraged to choose low-GI CHO and reduce saturated fat.<br>Duration: 9 weeks	Int: 7.2±4.6<br>Con: 7.8±4.6<br>$p = 0.04$<br>GWG: Pre-pregnancy – delivery	Random: Low<br>AC: Unclear<br>Blinding: Unclear<br>Data: Unclear<br>SR: Low<br>Other: Unclear

Ramirez-Velez[48]	Cali, Colombia<br>Mar 2008 –<br>Jan 2010	Nulliparous, 16-30 years, 16-20 weeks gestation, physically inactive.<br>Sample: 67<br>Final analyses: 24 int, 26 con	Supervised PA session, 60 mins, 3 days per week, individualised nutrition plan and a light breakfast before each PA session.<br>Duration: 12 weeks	Only reported weight at baseline and end.<br>*Not significant*<br>GWG: Baseline – end of intervention	Random: Unclear<br>AC: Unclear<br>Blinding: Unclear<br>Data: Low<br>SR: High<br>Other: Unclear
Rauh[107]	Munich, Germany<br>Feb 2010 –<br>Aug 2011	Singleton pregnancy, > 18 years old, < 18 weeks<br>gestation, ≤ 1 child.<br>Excluded: contraindications to PA, diabetes, uncontrolled chronic disease that may affect weight gain, psychiatric illness.<br>Sample: 250 (167 int, 83 con)<br>Final analyses: 152 int, 74 con	Two individual counselling sessions (at 20 and 30 weeks) regarding LS, self-monitoring, PA, weight and goal-setting.<br>Duration: 20 weeks	Int: 14.1±4.1<br>Con: 15.6±5.8<br>$p = 0.035$<br>GWG: Pre-pregnancy - delivery	Random: Unclear<br>AC: Unclear<br>Blinding: Unclear<br>Data: Low<br>SR: Unclear<br>Other: Low
Renault[49]	Copenhagen, Denmark<br>Mar 2009 –<br>Mar 2012	Singleton pregnancy, > 18 years, < 16 weeks gestation, obese.<br>Excluded: diabetes, serious diseases limiting PA, bariatric surgery, alcohol or drug abuse.<br>Sample: 425 (142 PA + Diet, 142 PA, 141 con)<br>Final analyses: 130 PA + Diet, 125 PA, 134 con	PA + Diet: PA and diet intervention. Women advised to increase PA to 11,000 steps/day. They also had contact with a dietitian every 2 weeks (alternating between outpatients and phone calls).<br>PA: PA intervention. Women advised to increase PA to 11,000 steps per day.<br>Duration: 26 weeks	PA + Diet: 8.6 (-9.6 - 34.1)[c]<br>PA: 9.4 (-3.4 – 28.2)[c]<br>Con: 10.9 (-4.4 – 28.7)[c]<br>$p < 0.01$ interventions and control.<br>GWG: Pre-pregnancy – 36/37 weeks	Random: Unclear<br>AC: Unclear<br>Blinding: Unclear<br>Data: Low<br>SR: Low<br>Other: Unclear
Ruchat[103]	Ontario, Canada	Singleton pregnancy, > 18 years, 16 – 20 weeks gestation, healthy weight.<br>Excluded: > 40 years, chronic, disease, contraindications to PA.<br>Sample: 94 (23 low, 26 mod, 45 con)<br>Final analyses: 23 low, 26 mod,	Low: Low intensity PA regimen and modified GDM diet based on 8360kJ/day, 40-55% CHO, 20-30% protein, 30% fat.<br>Mod: Moderate intensity PA regimen with the same dietary advice as low intensity.<br>Duration: 20-24 weeks	Low: 15.3±2.9<br>Mod: 14.9±3.8<br>Con: 18.3±5.3<br>$p < 0.01$ interventions and control.<br>GWG: Pre-pregnancy - delivery	Random: Unclear<br>AC: Unclear<br>Blinding: Unclear<br>Data: Low<br>SR: Unclear<br>Other: Low
Sagedal[110]	Norway<br>Sep 2009 -<br>Feb 2013	Singleton pregnancy, ≤20 weeks gestation, pre-pregnancy BMI ≥19 kg/m^2, literate in Norwegian or<br>English.<br>Excluded: diabetes, contraindication to PA, substance abuse.<br>Sample: 606 (303 int, 303 con)<br>Final analyses: 296 int, 295 con	Two phone consultations (baseline and 4 – 6 weeks later). Dietary advice included portion sizes, regular meals, snacking, increasing intake of water, fruits, and vegetables. Women also provided access to a gym, cooking class, and LS booklet.<br>Duration: 24 weeks	Int: 14.4±6.2<br>Con: 15.8±5.7<br>$p < 0.01$	Random: Low<br>AC: Low<br>Blinding: Low<br>Data: Low<br>SR: Low<br>Other: Low

Simmons[42]	Barcelona, Cambridge, Vienna, Leuven, Copenhagen, Poznan, Padova, Galway, Amsterdam, Jan 2012 – Aug 2013	Singleton pregnancy, ≥18 years, <20 weeks Gestation, pre-pregnancy BMI ≥29kg/m^2. Excluded: pre-existing diabetes, inability to walk ≥100m, requiring a complex diet, chronic medical condition, psychiatric illness Sample: 150 (50 HE, 50 PA, 50 HE + PA) Final analyses: 31 HE, 30 PA, 34 HE + PA	Five face-to-face sessions and 4 x optional phone sessions with either PA, HE or a combination of both. Duration: 25 weeks	HE: 7.6±5.3 PA: 9.6±4.3 HE + PA: 8.5±4.2 *HE vs HE+PA* $p = 0.39$ *HE vs PA* $p = 0.02$ *HE+PA vs PA* $p = 0.24$ GWG: Randomization - 37 weeks	Random: Low AC: Low Blinding: Low Data: High SR: Unclear Other: Unclear
Skouteris[109]	Melbourne, Australia Aug 2011 – Aug 2013	Singleton pregnancy, >18 years old, <18 weeks gestation. Sample: 261 (131 int, 130 con) Final analyses: 131 int, 130 con	Int: Individual and group health coaching regarding healthy LS for weight management, mood and body image. An initial individual session followed by two phone-calls and two group sessions regarding healthy behaviours. Con: Two 2-hour education sessions similar to the group sessions in the intervention group. Duration: 24 weeks	Int: 12.8±6.7 Con: 12.1±6.8 $p = 0.38$ GWG: Pre-pregnancy - delivery	Random: Low AC: Low Blinding: Low Data: Unclear SR: Unclear Other: Low
Thomson[43]	Lower Mississippi, United States Apr 2013 – May 2015	Singleton pregnancy, <19 weeks, >18 years, 1st – 3rd child. Excluded: NR Sample: 105 (54 PATE, 51 PAT) Final analyses (ITT): PATE 43, PAT 39	PAT: One-on-one home visits, monthly group meetings, developmental screenings and a resource network for families. PATE: PAT curriculum plus greater emphasis on healthy eating and GWG in pregnancy. Activities, DVDs and goal setting for diet and exercise. Duration: 22 weeks	PAT: 13.8 ±10.2 PATE: 10.9±8.7 *Significance not reported* GWG: Pre-pregnancy to delivery (both self-reported)	Random: Low AC: Unclear Blinding: Unclear Data: Unclear SR: High Other High
Vesco[113]	Oregon and Washington, United States Oct 2009 - July 2011	Singleton pregnancy, > 18 years, obese. Excluded: diabetes, medical conditions requiring nutrition care. Sample: 118 (58 int, 60 con) Final analyses: 56 int, 57 con	Two individual counselling sessions at baseline and weekly group sessions to assist women to maintain their weight within 3% of their baseline weight. Dietary advice was based on DASH diet. Women were advised to participate in 30 mins of moderate PA per day. Duration: 19-32 weeks	Int: 5.0±4.1 Con: 8.4±4.7 $p < 0.01$ GWG: Randomization - 34 weeks	Random: Low AC: High Blinding: Unclear Data: Low SR: Unclear Other: Low
Vinter[41]	Odense, Denmark Oct 2007 – Oct 2010	Singleton pregnancy, 18-40 years, 10-14 weeks gestation, obese. Excluded: chronic medical disorders, previous obstetric complications. Sample 360 (180 int, 180 con) Final analyses: 150 int, 154 con	Four dietary counselling sessions to limit GWG to 5kg. Energy based on BMI and 30-60 mins PA per day encouraged. Women provided with pedometer, gym membership, and one training session per week. Duration: 20 weeks	Int: 7.0 (4.7 – 10.6)[c] Con: 8.6 (5.7 – 11.5)[c] $p = 0.014$ GWG: 15 – 35 weeks	Random: Low AC: Low Blinding: Unclear Data: Low SR: Low Other: Low

Wang[50]	Peking, China<br>Sep 2012 - Jan 2013	Singleton pregnancy, at least one risk factor for GDM.<br>Excluded: diabetes.<br>Sample: 272 (134 int, 138 con)<br>Final analyses: Unclear	Three LS counselling sessions regarding diet, PA and GWG. Reinforcement of LS advice given accordingly.<br>Duration: 22 weeks	Early pregnancy<br>Int: 1.4±2.3<br>Con: 1.4±2.6<br>$p = 0.91$<br>Mid-pregnancy<br>Int: 5.5±2.2<br>Con: 5.7±2.3<br>$p = 0.57$	Random: Unclear<br>AC: Unclear<br>Blinding: Unclear<br>Data: Low<br>SR: High<br>Other: High

[a] Gestational weight gain (kg): mean (standard deviation) unless otherwise stated.

[b]Based on Cochrane Collaboration Risk of Bias. Random = Randomization; AC = Allocation concealment; Blinding = Blinding of participants, personal and outcome; Data = Incomplete data; SR = Selective reporting; Other = Other bias.

[c] GWG (kg) median and range

Other acronyms and abbreviations in alphabetical order: BMI = body mass index, Con = control group, DASH = dietary approaches to stop hypertension, EUC = enhanced usual care, GI = glycemic index, GDM = gestational diabetes mellitus, GWG = gestational weight gain, HE = healthy eating, Int = intervention group, IOM = Institute of Medicine, LS = lifestyle, Mod = moderate, PA = Physical Activity, PAT = Parents as teachers, PATE/PAT+ = Parents as teachers enhanced
