# Supplementary material for: Attenuating Pregnancy Weight Gain—What Works and Why: A Systematic Review and Meta-Analysis
Source: Nutrients. 2018 Jul 22;10(7):944. doi: 10.3390/nu10070944 (PMC6073617; doi:10.3390/nu10070944)
Supplement: Supplementary file 1 [file nutrients-10-00944-s001.zip › Table S6_ eHealth Interventions_Results Summary.pdf]

Table S6: Description of eHealth interventions aimed at preventing excessive gestational weight gain.

First author	Setting and date	Population	Intervention	GWG[a] and GWG measure	Quality[b]
Herring[117]	Philadelphia, United States 2013 – 2014	Singleton pregnancy, > 18 years old, < 20 weeks gestation, BMI 25-45kg/m² in first trimester,<br>African American Medicaid recipient, cell phone with text messaging, Facebook account.<br>Excluded: Requiring specialised nutrition care, tobacco use.<br>Sample: 66 (33 int, 33 con)<br>Final analyses: 27 int, 29 con	Promotion of behaviour change goals regarding energy intake, PA and self-weighing. Skills training<br>and support were delivered using self-monitoring texts with personalised feedback, a closed Facebook group, and health coach calls throughout pregnancy.<br>Duration: 28 weeks	Int: 8.7±6.6<br>Con: 12.3±6.4<br>$p < 0.05$<br>GWG: Early pregnancy - delivery	Random: Low<br>AC: Low<br>Blinding: Unclear<br>Data: Low<br>SR: Unclear<br>Other: Low
Jackson[121]	San Francisco, United States	Pregnant women, > 18 years old, < 26 weeks gestation.<br>Sample: 327 (158 int, 163 con)<br>Final analyses: 134 int 153 con	A computerised Video Doctor program in a clinical setting as part of routine prenatal care. It included<br>in-depth behavioural risk assessments, tailored counselling messages for both patient and clinician, a cueing sheet for the clinician, and worksheets<br>Duration: 4 weeks	Int: 15.1 (SD NR)<br>Con: 15.2 (SD NR)<br>$p = 0.95$<br>GWG: Pre-pregnancy - delivery	Random: Unclear<br>AC: Unclear<br>Blinding: Unclear<br>Data: Low<br>SR: Unclear<br>Other: Unclear
Olsen[119]	United States May 2011 – Jul 2012	Singleton pregnancy, < 20 weeks, 18-35 years.<br>Excluded: BMI < 18.5 and >35, weight-affecting medications, no email.<br>Sample: 1689<br>Final analyses: 1126 int, 563 con	Int: Website with blogs, local resources, GWG trackers, PA goal setting tools and weekly emails.<br>Con: Access to control website with general pregnancy advice.<br>Duration: 28 weeks.	Int: 47.6% exceeded IOM<br>Con: 45.9% exceeded IOM<br>*Not significant*<br>GWG: First weight < 14 weeks – 37 weeks	Random: Low<br>AC: Low<br>Blinding: Unclear<br>Data: High<br>SR: High<br>Other: High
Smith[120]	United States Jan 2013 – Sept 2013	18-45 years, relatively inactive, regular Internet access.<br>Excluded: History of GDM, pre-eclampsia, chronic disease, underweight, smoker, metabolic condition.<br>Sample: 51 (26 int, 25 con)<br>Final analyses: 24 int, 21 con	Web-based PA advice with information about goal-setting, problem solving, PA diary and forum. Website tutorial also provided.<br>Duration: 30 weeks	Int: 13.6±5.6<br>Con: 11.2±5.1<br>*Not significant*<br>GWG: Pre-pregnancy to 36 weeks	Random: Low<br>AC: Unclear<br>Blinding: Unclear<br>Data: Low<br>SR: High<br>Other: High
Willcox[118]	Australia May 2014 – Oct 2014	Singleton pregnancy, 10-17 weeks, BMI > 25, English speaking, mobile phone.<br>Excluded: < 18 years, comorbidities requiring additional management.<br>Sample: 100 (50 int, 50 con)<br>Final analyses: 45 int, 46 con	Multi-faceted mHealth intervention to promote healthy diet, PA and GWG. Intervention booklet, text messages, website, video messages and Facebook were used.<br>Duration: 21 weeks	Int: 7.8±4.7<br>Con: 9.7±3.9<br>$p = 0.04$<br>GWG: Baseline – 36 weeks	Random: Low<br>AC: Low<br>Blinding: Unclear<br>Data: Low<br>SR: Low<br>Other: Unclear

[a] Gestational weight gain (kg): mean (standard deviation) unless otherwise stated.
[b]Based on Cochrane Collaboration Risk of Bias. Random = Randomization; AC = Allocation concealment; Blinding = Blinding of participants, personal and outcome; Data = Incomplete data; SR = Selective reporting; Other = Other bias.
Other acronyms and abbreviations in alphabetical order: BMI = body mass index, Con = control group, GI = glycemic index, GWG = gestational weight gain, HTN = hypertension, Int = intervention group, IOM = Institute of Medicine, NR = Not reported, PA = Physical Activity.
