# Supplementary material for: Attenuating Pregnancy Weight Gain—What Works and Why: A Systematic Review and Meta-Analysis
Source: Nutrients. 2018 Jul 22;10(7):944. doi: 10.3390/nu10070944 (PMC6073617; doi:10.3390/nu10070944)
Supplement: Supplementary file 1 [file nutrients-10-00944-s001.zip › Table S7_ Other Interventions_Results Summary.pdf]

Table S7: Description of 'other' interventions aimed at preventing excessive gestational weight gain (other than diet, physical activity, lifestyle and eHealth).

First author	Setting and date	Population	Intervention	GWG[a] and GWG measure	Quality[b]
Bechtel-Blackwell[127]	United States	African American, 13-18 years old, primiparous.<br>Sample: 60 (30 int, 30 con)<br>Final analyses: 22 int, 24 con	Three, 20 minute group sessions regarding nutrition needs specific to stage of pregnancy and nutrition assessment using computer assisted self-interviewing.<br>Duration: 22-24 weeks	Trimester 1: Int:1.5 ; Con:2.8[c]<br>$p < 0.01$<br>Trimester 2: Int:6.6; Con:6.8[c]<br>$p > 0.05$<br>Trimester 3: Int:6.9; Con:5.6[c]<br>$p < 0.01$<br>GWG: Per trimester	Random: High<br>AC: High<br>Blinding: Unclear<br>Data: High<br>SR: Unclear<br>Other: Unclear
Brownfoot[30]	Melbourne, Australia<br>Jan 2010 – Nov 2012	Singleton pregnancy, < 21 weeks gestation, antenatal care through hospital clinic.<br>Excluded: <18 years, >45 years, medical comorbidities, substance abuse, inability to speak English.<br>Sample: 782 (386 int, 396 con)<br>Final analyses: 369 int, 372 con	Women were weighed at each antenatal visit. All clinic rooms had the IOM guidelines displayed and clinicians were encouraged to discuss appropriate GWG with patients.<br>Duration: 22 weeks	Int: 0.54±0.28[d]<br>Con: 0.53±0.24[d]<br>$p = 0.63$<br>GWG: Baseline - final visit	Random: Unclear<br>AC: Low<br>Blinding: Unclear<br>Data: Low<br>SR: Low<br>Other: Low
Daley[122]	United Kingdom<br>Apr 2012 – Dec 2012	Singleton pregnancy, > 18 years old, BMI 18-29.9kg/m^2.<br>Excluded: Obesity, pregnancy complications.<br>Sample: 76 (40 int, 36 con)<br>Final analyses: 34 int, 34 con	A midwife-led intervention as part of usual antenatal care. Women were weighed at each appointment and weights were plotted on GWG chart. Women were also given a weight record chart and asked to monitor their own progress between visits.<br>Duration: 26-28 weeks	Int: 12.0±4.5<br>Con: 12.1±5.9<br>*Significance not reported*<br>GWG: Baseline - 38 weeks	Random: Unclear<br>AC: Unclear<br>Blinding: Unclear<br>Data: Low<br>SR: Unclear<br>Other: Low
Herrera-Perdigon[126]	Philadelphia, United States<br>Jan 1992 - Jan 1996	High-risk pregnancy (Pre-GDM, GDM, HTN).<br>Excluded: < 18 years, risk of pre-term labour, multiple pregnancy.<br>Sample: 77<br>Final analyses: 29 int, 38 con	Usual physician-led care was modified to so half prenatal visits were with a nurse at home. Visits with physician or nurse were every 1-2 weeks. Advice regarding nutrition and lifestyle provided.<br>Duration: 17 weeks	Int: 11.8±8.5<br>Con: 14.2±10.3<br>$p = 0.91$<br>GWG: Pre-pregnancy - delivery	Random: Unclear<br>AC: Low<br>Blinding: Unclear<br>Data: Low<br>SR: Unclear<br>Other: Unclear
Jeffries[123]	Melbourne, Australia<br>Jul 2007 – May 2008	Singleton pregnancy, 18-45 years old.<br>Excluded: pre-existing diabetes, non-English speaking<br>Sample: 286 (148 int, 138 con)<br>Final analyses: 125 int, 138 con	Education regarding GWG guidelines.<br>Women's weights were recorded at 16, 20, 24, 28, 30, 32 and 34 weeks.<br>Duration: 25 weeks	Int: 10.7±4.2<br>Con: 11.5±4.0<br>$p = 0.42$<br>GWG: Baseline - 36 weeks	Random: Low<br>AC: Low<br>Blinding: Unclear<br>Data: Low<br>SR: Unclear<br>Other: Low

Quinlivan[124]	Melbourne, Australia	Singleton pregnancy, overweight or obese.<br>Excluded: foetal abnormalities, wanted to relinquish infant after birth, unable to attend clinic, non-English speaking.<br>Sample: 132 (67 int, 65 con)<br>Final analyses: 63 int, 61 con	An intervention with four key aspects: a) Continuity of care, b) Assessing weight gain at each antenatal visit, c) Dietary intervention by a food technologist at each antenatal visit, d) Psychological assessment and intervention if indicated<br>Duration: 30 weeks	Int: 7.0±5.2<br>Con: 13.8±5.2<br>$p < 0.01$<br>GWG: Baseline - delivery	Random: Low<br>AC: Low<br>Blinding: Unclear<br>Data: Unclear<br>SR: Unclear<br>Other: Unclear
Santamaria[125]	Italy<br>Jan 2012 –<br>Jul 2013	Singleton pregnancy, pre-pregnancy BMI >25 and <30, first trimester fasting plasma glucose <126mg/dl and or random glycaemia <200mg/dl, Caucasian.<br>Excluded: Previous GDM, pre-GDM, corticosteroids.<br>Sample: 220 (110 int, 110 con)<br>Final analyses: 95 int, 102 con	2g myo-inositol + 200ug folic acid twice/day.<br>Duration: 12 weeks	Int: 6.2±3.2<br>Con: 7.5±4.0<br>$p = 0.07$<br>GWG: Baseline – Oral glucose tolerance test.	Random: Low<br>AC: Low<br>Blinding: Unclear<br>Data: Low<br>SR: Unclear<br>Other: Unclear
Syngelaki[29]	London,<br>United Kingdom<br>Oct 2010 –<br>Jun 2013	Singleton pregnancy, 12-18 weeks gestation, BMI >35kg/m^2.<br>Excluded: <18 years old, major foetal defect detected, history of GDM, kidney, heart or liver failure, hyperemesis gravidarum, prescribed metformin before screening, sensitivity to metformin.<br>Sample: 450 (225 int, 225 con)<br>Final analysis: 202 int, 198 con	Metformin was initiated at an initial dose of 1g per day and increased 0.5g per week to a maximum dose of 3g per day in week five. Both control and intervention groups received advice regarding healthy eating and low-GI foods, and were encouraged to exercise 30 mins per day.<br>Duration: 25 weeks	Int: 4.6 (1.3-7.2)[e]<br>Con: 6.3 (2.9-9.2)[e]<br>$p < 0.001$<br>GWG: Randomization - delivery	Random: Low<br>AC: Low<br>Blinding: Low<br>Data: Unclear<br>SR: Unclear<br>Other: Unclear

[a] Gestational weight gain (kg): mean (standard deviation) unless otherwise stated.
[b]Based on Cochrane Collaboration Risk of Bias. Random = Randomization; AC = Allocation concealment; Blinding = Blinding of participants, personal and outcome; Data = Incomplete data; SR = Selective reporting; Other = Other bias.
[c]GWG per trimester (SD not reported).
[d]Weekly rate of GWG (kg/week).
[e]GWG (kg) median and interquartile range.
Other acronyms and abbreviations in alphabetical order: BMI = body mass index, Con = control group, GI = glycemic index, GWG = gestational weight gain, HTN = hypertension, Int = intervention group, IOM = Institute of Medicine, NR = Not reported, PA = Physical Activity.
