# Supplementary material for: Attenuating Pregnancy Weight Gain—What Works and Why: A Systematic Review and Meta-Analysis
Source: Nutrients. 2018 Jul 22;10(7):944. doi: 10.3390/nu10070944 (PMC6073617; doi:10.3390/nu10070944)
Supplement: Supplementary file 1 [file nutrients-10-00944-s001.zip › Table S8_Sensitivity analyses_High risk removed.pdf]

Table S8: Random-effects meta-analyses for diet, physical activity, lifestyle and eHealth interventions (WMD and 95% CI), studies with a high risk of bias removed.

	Sub-group and overall	Studies[a]	Participants	Overall effect size WMD (95% CI)	p value	I^2
Diet	**Overall: Measurement GWG > 24 weeks**	**8**	**1949**	**-3.25 (-5.11, -1.40)**	**<0.00**	**93.5%**
Physical activity	Measurement GWG 12-24 weeks	2	215	-1.23 (-4.66, 2.19)	0.48	90.2%
	Measurement GWG > 24 weeks	15	3134	-0.99 (-1.66, -0.32)	<0.00	84.7%
	**Overall**	**17**	**3349**	**-1.04 (-1.70, -0.38)**	**<0.00**	**85.4%**
Lifestyle	Measurement GWG 12-24 weeks	3	2908	-0.37 (-0.95, 0.21)	0.21	42.9%
	Measurement GWG > 24 weeks	18	4371	-0.83 (-1.38, -0.28)	<0.00	68.5%
	**Overall**	**21**	**7279**	**-0.73 (-1.17, -0.29)**	**<0.00**	**65.7%**
eHealth	**Overall: Measurement GWG > 24 weeks**	**2**	**147**	**-2.26 (-3.84, -0.69)**	**<0.00**	**0.00%**

[a] Some studies included sub-groups as no overall GWG was reported.
