# Supplementary figures and images for: Attenuating Pregnancy Weight Gain—What Works and Why: A Systematic Review and Meta-Analysis
Source: Nutrients. 2018 Jul 22;10(7):944. doi: 10.3390/nu10070944 (PMC6073617; doi:10.3390/nu10070944)

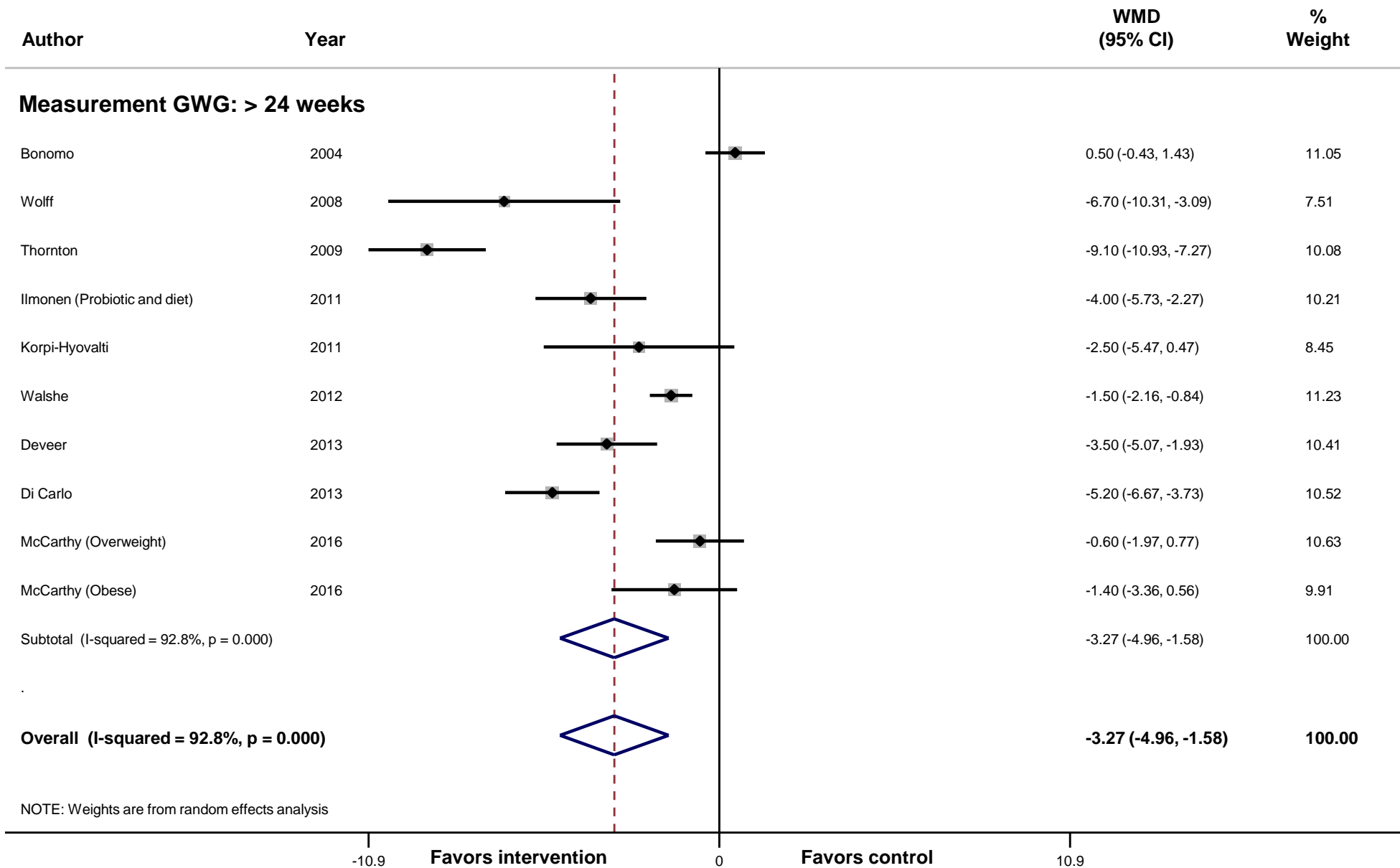

Supplement: Supplementary file 1 [file nutrients-10-00944-s001.zip › Figure S1_Diet Meta-analysis.pdf]

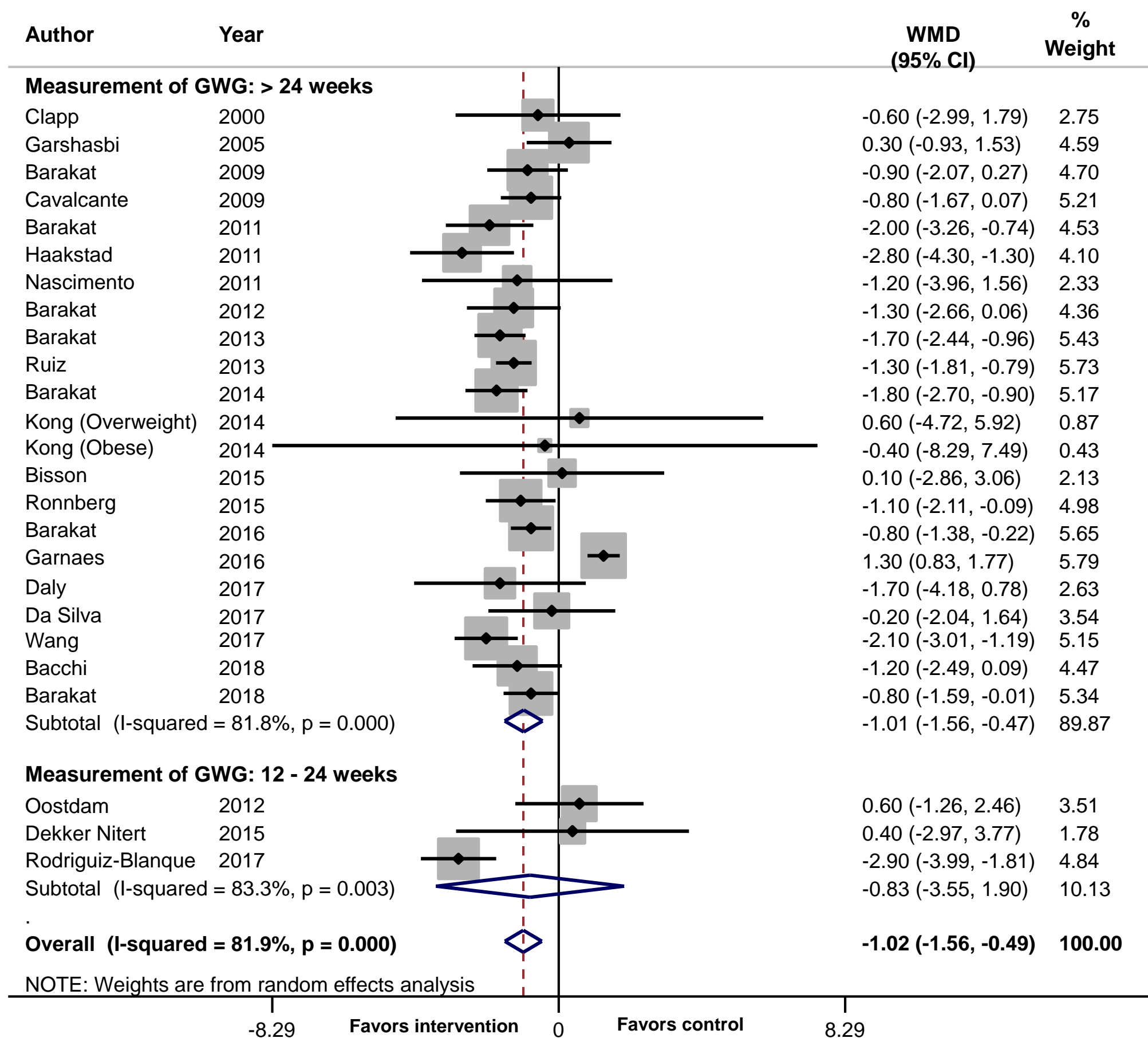

Supplement: Supplementary file 1 [file nutrients-10-00944-s001.zip › Figure S2_PA Meta-analysis.pdf]

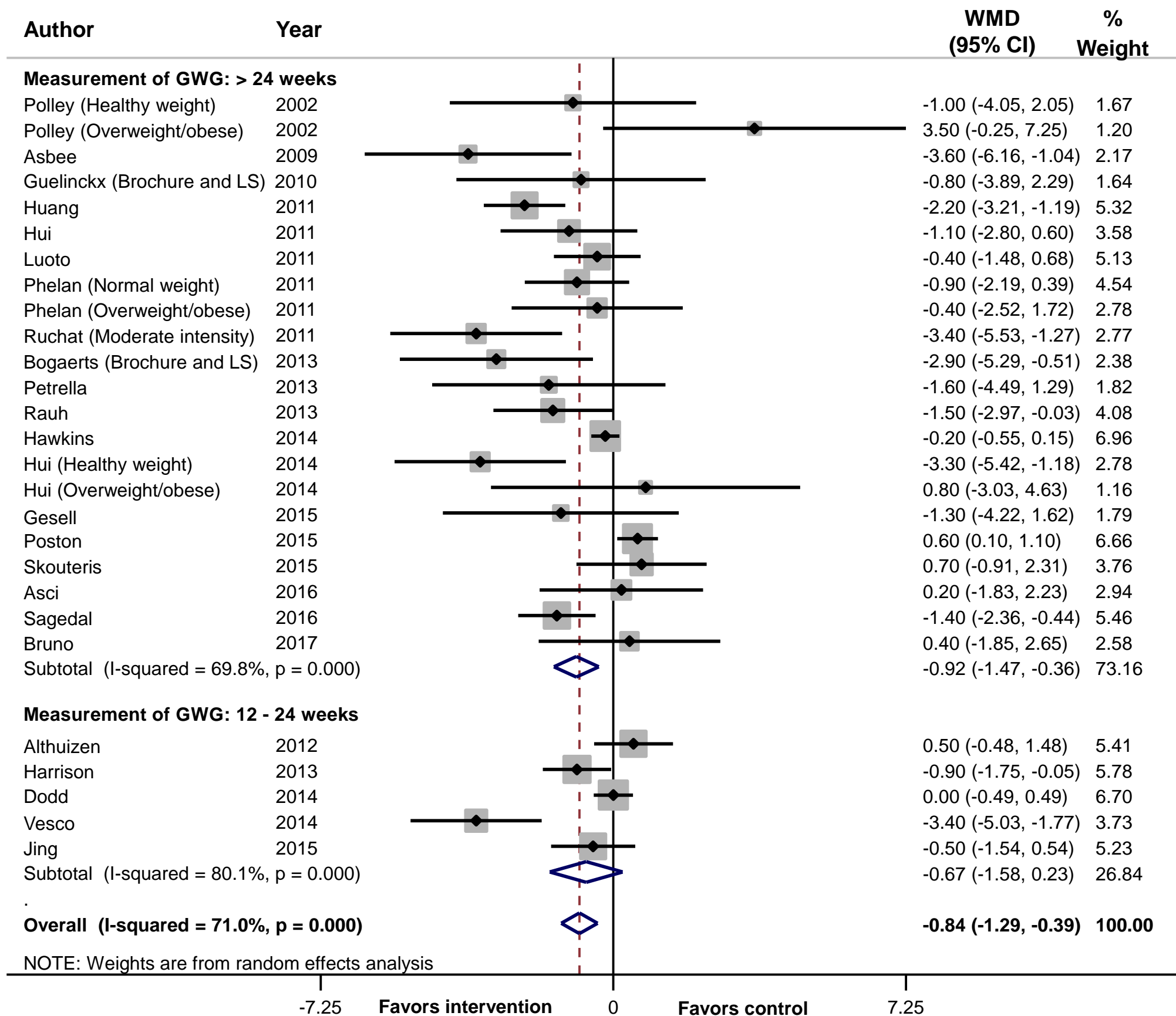

Supplement: Supplementary file 1 [file nutrients-10-00944-s001.zip › Figure S3_LS Meta-analysis.pdf]

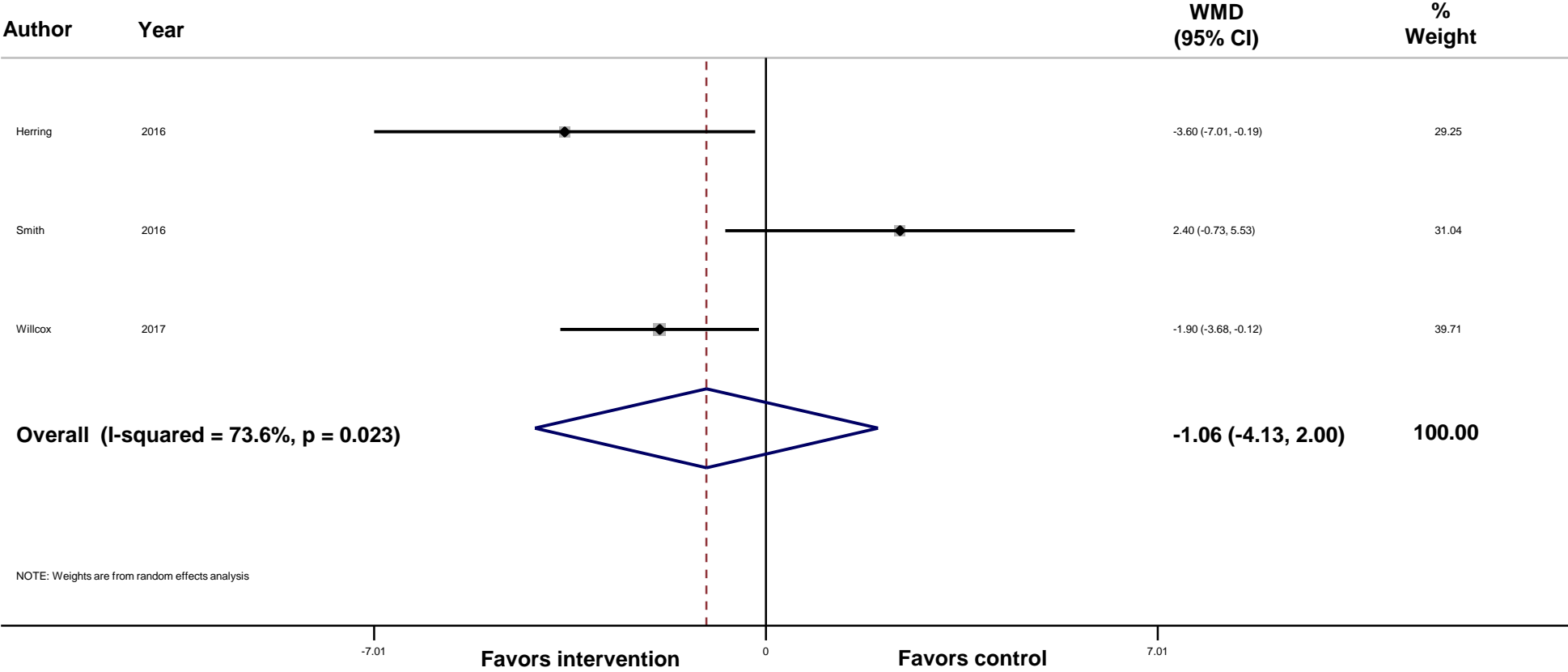

Supplement: Supplementary file 1 [file nutrients-10-00944-s001.zip › Figure S4_eHealth Meta-analysis.pdf]

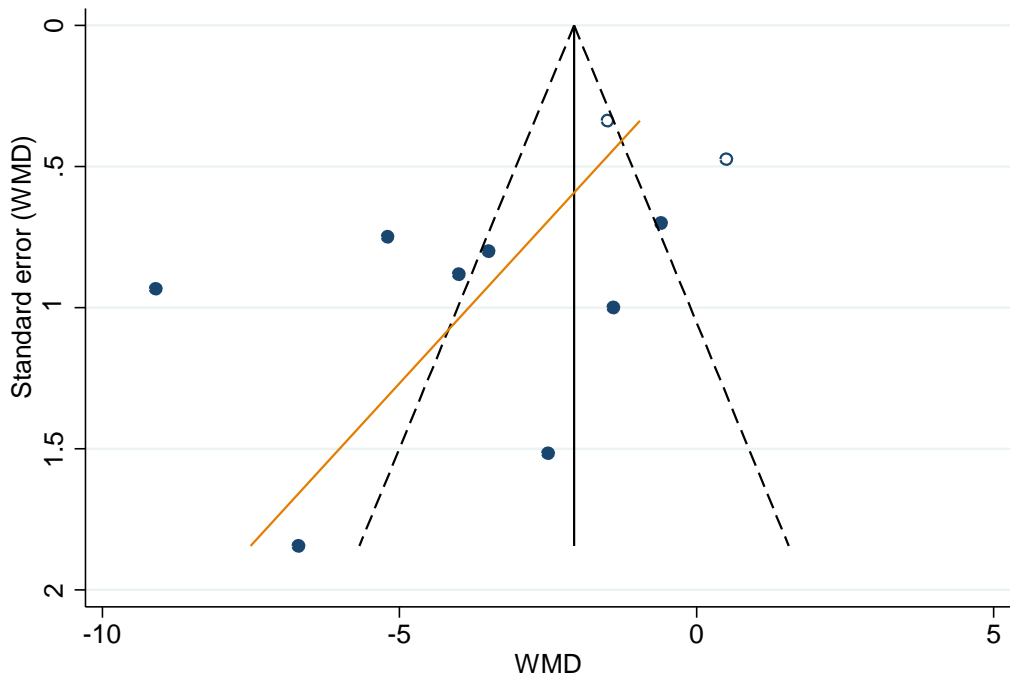

Supplement: Supplementary file 1 [file nutrients-10-00944-s001.zip › Figure S5_Diet Funnel Plot.pdf]

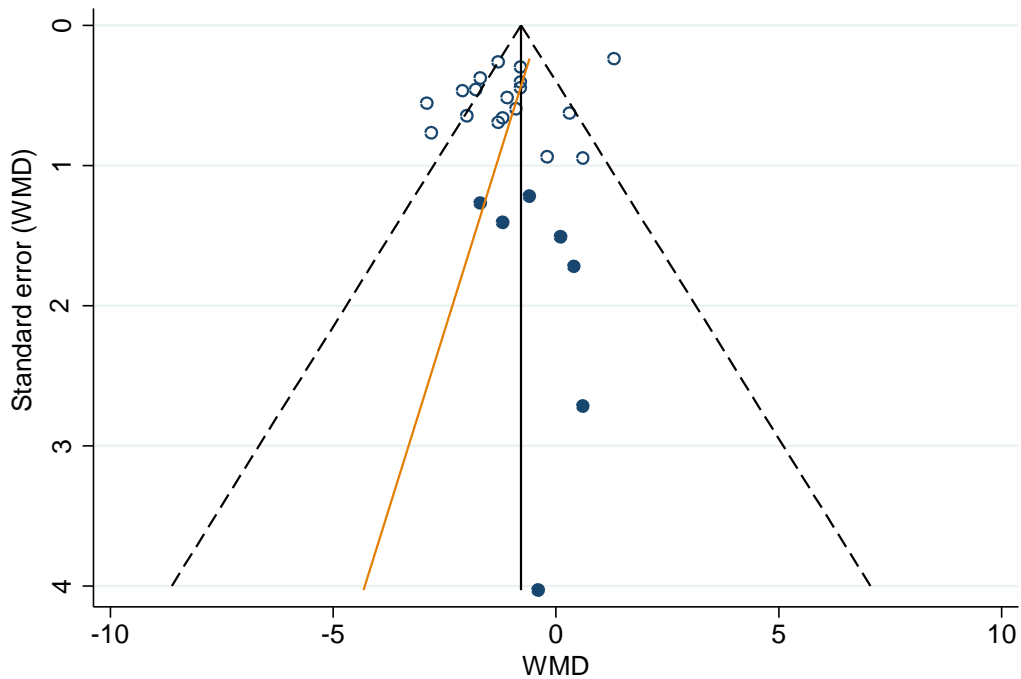

Supplement: Supplementary file 1 [file nutrients-10-00944-s001.zip › Figure S6_PA Funnel Plot.pdf]

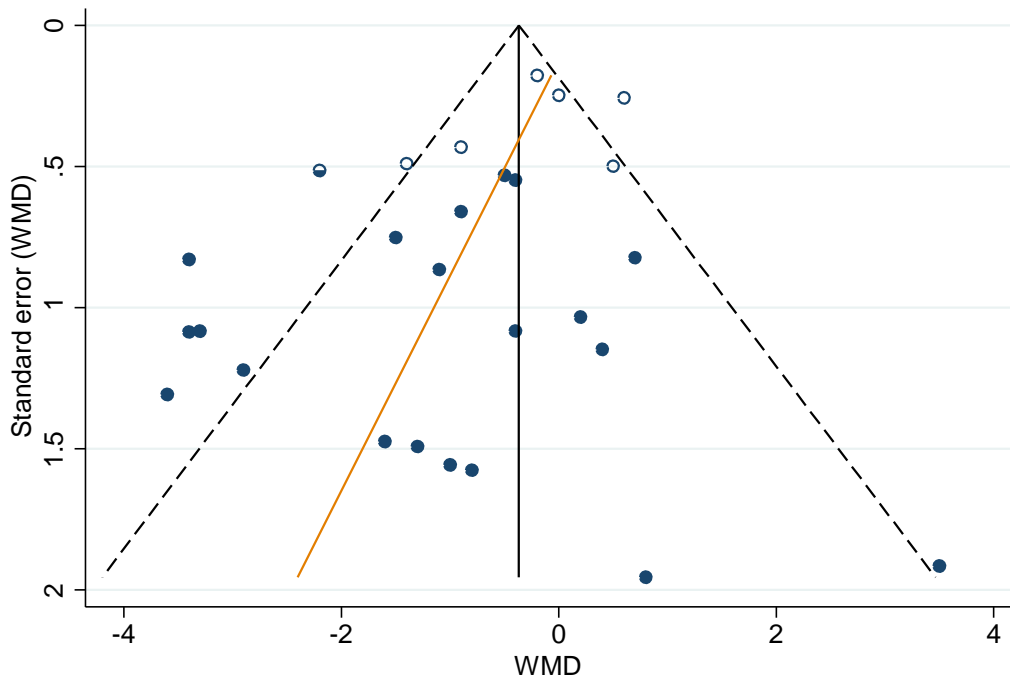

Supplement: Supplementary file 1 [file nutrients-10-00944-s001.zip › Figure S7_LS Funnel Plot.pdf]

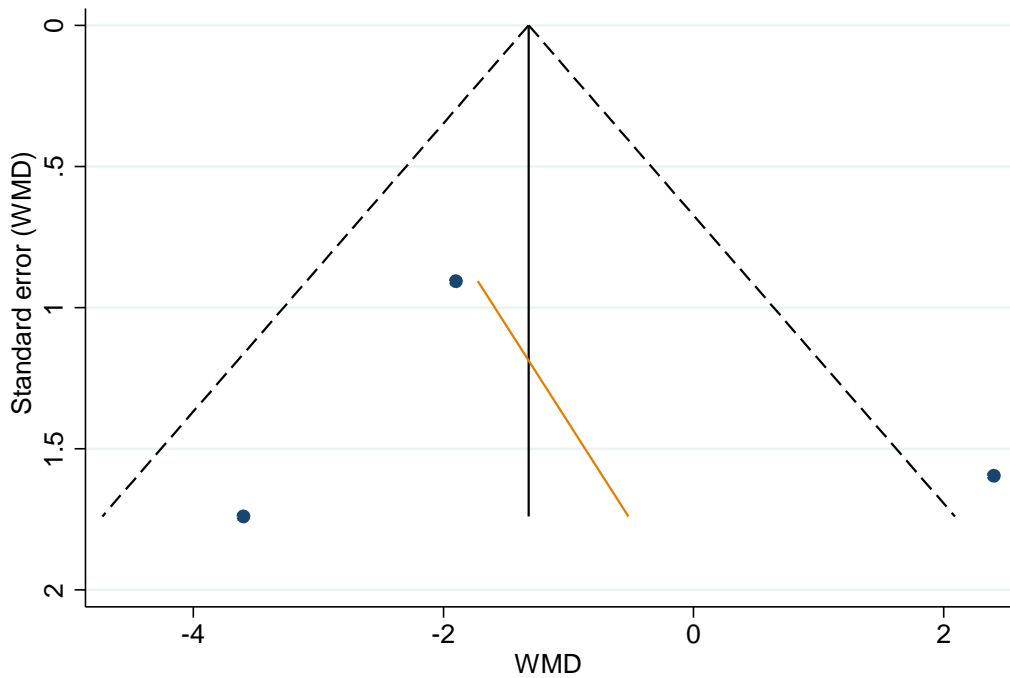

Supplement: Supplementary file 1 [file nutrients-10-00944-s001.zip › Figure S8_eHealth Funnel Plot.pdf]
